# Exposure to low concentrations of PM_2.5_ and its constituents with preterm birth in Shenzhen, China: a retrospective cohort study

**DOI:** 10.1186/s12889-025-22489-7

**Published:** 2025-04-07

**Authors:** Minting Zhu, Zhongai Ouyang, Tao Liu, Weigui Ni, Zhijian Chen, Bingyi Lin, Lijuan Lai, Yi Jing, Long Jiang, Jingjie Fan

**Affiliations:** 1 School of Public Health, Southern Medical University, No.1023-1063, Shatai South Road, Baiyun District, Guangzhou, 510515 China; 2 Department of Preventive Healthcare, Shenzhen Maternity and Child Healthcare Hospital, Southern Medical University, No.2004 Hongli Road, Futian District, Shenzhen, 518028 China; 3 Department of Public Health and Preventive Medicine, School of Medicine, Jinan University, Guangzhou, 510632 China; 4 China Greater Bay Area Research Center of Environmental Health, School of Medicine, Jinan University, Guangzhou, 510632 China

**Keywords:** Air pollution, PM_2.5_, Chemical constituents, Preterm birth, Cohort study

## Abstract

**Background:**

Due to the Air Pollution Prevention and Control Measures issued by the Chinese government, air quality has significantly improved, particularly with respect to PM_2.5_. However, studies on the relationship between low concentrations of PM_2.5_ and preterm birth (PTB) remain limited in China.

**Objective:**

To examine the associations between low concentrations of PM_2.5_ and its constituents and PTB.

**Methods:**

This retrospective cohort study was conducted from July 2021 to April 2023 in Shenzhen, China. Data on questionnaires and pregnancy outcomes were collected for each participant. Using the Tracking Air Pollution in China (TAP) dataset, we assessed the concentrations of PM_2.5_ and its chemical constituents, including sulfate (SO_4_^2−^), nitrate (NO_3_^−^), organic matter (OM), black carbon (BC), and ammonium (NH_4_^+^). We applied a generalized additive model (GAM) to evaluate the relationship. The relationship between exposure to PM_2.5_ and its constituents and PTB was further examined using a method that combined dummy variable settings with trend tests. Stratified analysis was conducted to explore the potential factors.

**Results:**

Among 17,240 live-born infants, the rate of PTB was 6.0%, and the average exposure concentration of PM_2.5_ was 20.24 μg/m^3^. There were positive associations between PM_2.5_ and its constituents and PTB. With each interquartile range (IQR) increase in PM_2.5_ during the third trimester, the risk of PTB increased by 2.23 times. The exposure effects of sulfate (SO_4_^2−^) and organic matter (OM) were comparable to the total PM_2.5_. The third trimester might be the critical susceptibility window. The risk was higher among women who conceived in the cold season and were exposed to higher temperatures during pregnancy.

**Conclusion:**

Even at low levels, PM_2.5_ can still increase the risk of PTB, with varying health effects attributed to different constituents. This underscores the importance of further strengthening environmental management and characterizing the contributions of PM_2.5_ sources.

**Supplementary Information:**

The online version contains supplementary material available at 10.1186/s12889-025-22489-7.

## Background

Preterm birth (PTB) is defined as birth before 37 weeks of gestation [1]. PTB affects not only the growth and development of infants after birth [2–5] but also increases the future risk of hypertension, stroke, and ischemic heart disease in mothers in the future [6–9]. An estimated 13.4 million newborn babies were born PTB in 2020, with China ranking fourth in the world after India, Pakistan, and Nigeria. Although the number of PTBs appears to have decreased compared to 2010, the overall PTB rate has not declined [10]. The risk of PTB is heightened by a variety of factors, including a history of adverse pregnancies, unhealthy lifestyle habits, and lower socioeconomic status [11–13]. In recent years, the impact of air pollution on adverse pregnancy outcomes, such as PTB, has become increasingly significant. Statistics on the burden of PTB due to environmental pollution in China suggest that 23% of PTBs can be attributed to environmental factors [14]. Air pollution, as a relatively controllable risk factor, has significant public health implications for understanding its relationship with maternal and infant health.

Previous studies in China have demonstrated an association between air pollution and PTB. However, these studies were primarily based on higher levels of PM_2.5_ exposure (> 35 μg/m^3^, the national annual secondary concentration limit) [15–18]. For instance, a study conducted in the Huai River Basin of Henan Province, China, which assessed the impact of particulate matter on adverse birth outcomes, found that PM_2.5_ exposure during the second trimester was associated with PTB and low birth weight, with an average PM_2.5_ exposure concentration of 99.0 μg/m^3^ [16]. Similarly, a multicenter cohort study in China reported that PM_2.5_ exposure during pregnancy was linked to an increased risk of PTB, with an average PM_2.5_ exposure concentration of 57.2 μg/m^3^ [19]. However, since 2013, China has implemented stringent air pollution control policies to safeguard public health, leading to a rapid decline in the concentrations of various air pollutants, particularly PM_2.5_ [20–23]. According to statistics, the population-weighted concentration of PM_2.5_ decreased from 63 μg/m^3^ in 2013 to 33 μg/m^3^ in 2023 [24]. The association between PM_2.5_ exposure and adverse pregnancy outcomes under improved air quality conditions remains unclear. Emerging evidence suggests that even low-concentration air pollution exposure can exert adverse health effects, indicating the absence of a safe threshold for air pollution [25, 26]. However, earlier large-scale studies from Western countries reported weak, null, or even inverse associations between PM_2.5_ exposure (below 35 μg/m^3^) and PTB [27–30]. In contrast, a study from Australia found that exposure to low-level PM_2.5_ was still associated with an increased risk of PTB [31]. The relationship between low-concentration air pollution exposure and adverse pregnancy outcomes remains inconclusive, which may be attributed to differences in regional air pollution conditions (e.g., sources, concentrations, and composition), exposure assessment methods, definitions of pregnancy exposure windows, and the demographic and geographic characteristics of the participants [17, 32–35]. Therefore, against the backdrop of declining global fertility rates, a deeper understanding of the association between improved air quality and maternal and child health is of great importance for evaluating past policies and guiding future air pollution control strategies.

Previous studies have confirmed an association between PM_2.5_ exposure and PTB. However, their findings exhibit variability, which may be largely attributed to differences in the chemical composition of PM_2.5_. As a complex mixture, PM_2.5_ consists of various constituents, including sulfate, nitrate, black carbon, and others. The toxicity of PM_2.5_ constituents varies significantly depending on their chemical composition, resulting in diverse and potentially distinct health impacts [33, 36, 37]. Although associations between PM_2.5_ constituents and PTB have been identified, the applicability of these findings is constrained by the geographical focus on developed countries and the lack of consensus on critical exposure periods [15, 35, 38, 39]. Given the differences in population susceptibility, economic conditions, and pollution profiles across regions, these findings may not be directly applicable to China under its current air quality improvements. Therefore, it is essential to investigate the association between exposure to low concentrations of PM_2.5_ constituents and adverse pregnancy outcomes. Such research will enhance our understanding of the toxic effects of PM_2.5_ and provide valuable insights for local emission control strategies.

This study, based on a birth cohort database, employs a retrospective cohort study design to assess the associations of exposure to low concentrations of PM_2.5_ and its constituents with the risk of PTB. Additionally, stratified analyses will be conducted to explore the potential factors influencing the relationship between PM_2.5_ and its constituents and PTB. We aim for our findings to provide targeted scientific evidence for environmental governance strategies in the context of China’s evolving air pollution landscape.

## Methods and materials

### Study design and participants

**Fig. 1:**
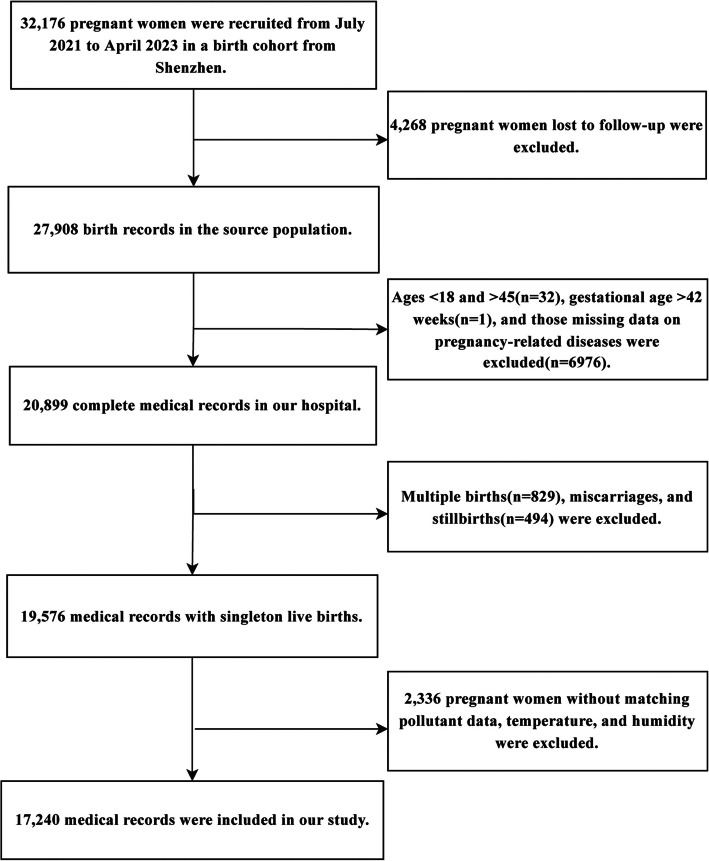
The flowchart for participant inclusion and exclusion

Using a birth cohort established from a top-tier maternity and child health hospital in Shenzhen, we conducted a retrospective cohort study to collect survey questionnaires and information on pregnancy outcomes. By integrating the exposure concentrations of PM_2.5_ and its constituents for each participant, a generalized additive model (GAM) was used to explore the relationship between PM_2.5_ and its constituent exposure during each trimester and the entire pregnancy with PTB, aiming to identify sensitive exposure windows and the primary constituents influencing PTB. Additionally, stratified analyses were conducted to examine the relationship between PM_2.5_ and its constituents and PTB, with a focus on identifying potential modifiers related to the third trimester. The participants were derived from a birth cohort established by a top-tier maternal and child health hospital in Shenzhen. Initially, we included the enrollment records of 32,176 pregnant women during the period from July 2021 to April 2023. According to the study design, we included pregnant women aged 18–45 who delivered before 42 weeks of gestation, excluding those with twins, miscarriages, and stillbirths. Additionally, we excluded pregnant women with missing PM_2.5_ and meteorological data (temperature, humidity), as well as those missing data on pregnancy-related diseases. The details of the exclusion criteria are illustrated in Fig. 1. Ultimately, a total of 17240 participants were included in our study. Informed consent was obtained from all participants at enrollment. The study protocol was approved by the Ethics Committee of Shenzhen Maternity and Child Healthcare Hospital (Ethical Approval Number: SFYLS[2022]032).

### Baseline investigation

The questionnaire information and pregnancy outcomes were collected, as detailed in Supplementary Table S1 and Table S2. The questionnaire information includes demographic characteristics of pregnant women and their husbands; pregnancy conditions; lifestyle habits; maternal medical conditions; and other relevant factors. Birth outcomes were obtained from medical records, including baseline delivery information and maternal medical conditions during pregnancy. Based on previous studies [40], we depicted the potential confounders using a Directed Acyclic Graph (DAG), as shown in Figure S1.

### Outcome definition

PTB is defined as a gestation period of less than 37 weeks [1]. The last menstrual period (LMP) was recorded by a trained obstetrician during the early pregnancy visit (gestational age < 14 weeks). The gestational age was calculated from the first day of the LMP until the date of delivery. The gestational age was re-adjusted by the obstetrician at the time of delivery. If there is a discrepancy between the two records, the corrected record was considered.

### PM_2.5_ and its constituents, temperature and relative humidity assessment

We defined four exposure periods for each birth: (1) the entire gestational period (from LMP to delivery), (2) the first trimester (< 14 weeks of pregnancy), the second trimester (14–28 weeks of pregnancy), and the third trimester (≥ 28 weeks to delivery).

PM_2.5_ is a complex mixture composed of sulfate, nitrate, ammonium salts, organic compounds, and other chemical constituents [41]. Previous epidemiological studies have shown that exposure to PM_2.5_ and its constituents during pregnancy is associated with PTB [42], with potential biological mechanisms including oxidative stress and inflammatory responses [43, 44]. Different PM_2.5_ constituents vary in toxicity and have differential health impacts [33, 36, 37]. Clarifying the associations between exposure to specific PM_2.5_ constituents and PTB can enhance our understanding of PM_2.5_ toxicity mechanisms and provide evidence-based insights for local emission control strategies.

Organic matter (OM) is composed of humic-like substances (HULIS), carboxylic acids, and sugar alcohols, among others, and is primarily sourced from aqueous processes, biogenic secondary organic aerosols (SOAs), biomass burning, mixed urban aerosols, and soil dust [45]. Data on PM_2.5_ and its chemical constituents, including sulfate (SO_4_^2−^), ammonium (NH_4_^+^), black carbon (BC), nitrate (NO_3_^−^), and OM, are derived from Tracking Air Pollution in China (TAP, http://tapdata.org.cn/). According to TAP, the concentration of OM can be estimated as 1.6 times the concentration of organic carbon (OC) [46].

The TAP project dataset has been widely employed in numerous high-quality epidemiological studies [47–49]. Utilizing a two-stage machine learning model, the TAP project integrates small-sample sampling techniques and a tree-based gap-filling method to estimate daily PM_2.5_ mass concentrations across China at a spatial resolution of 10 km. The out-of-bag cross-validation (CV) for this model yielded a coefficient of determination (R^2^) ranging from 0.80 to 0.88 and a root-mean-square error (RMSE) between 13.9 and 22.1 μg/m^3^ [50]. Based on the dataset with a spatial resolution of 10 km, the total mass concentration of PM_2.5_ in the TAP dataset was partitioned using conversion factors (CFs) simulated by the optimized Congestion Mitigation and Air Quality (CMAQ) model, with PM_2.5_ concentration as the overall constraint. The daily constituent concentrations of PM_2.5_ were evaluated, with correlation coefficients (R) ranging from 0.64 to 0.75 and RMSE values between 2.35 and 12.67 μg/m^3^ [46]. Based on the aforementioned datasets, we extracted the daily concentrations of PM_2.5_ and its chemical constituents from the grid cells corresponding to the residential addresses of the participants. We calculated the average exposure levels of PM_2.5_ and its chemical constituents for each participant across each trimester and the whole pregnancy.

The daily average temperature data in this study were derived from the 2-m air temperature in the ERA5 dataset, while the relative humidity data were calculated by analyzing the 2-m air temperature and 2-m dew point temperature data from the same dataset. The ERA5 dataset, created by the European Centre for Medium-Range Weather Forecasts (ECMWF), features a high spatial resolution of 0.1° × 0.1° [51]. The dataset integrates global observational data, advanced numerical models, and physical parameterization schemes. By employing data assimilation techniques and numerical simulation methods, it provides a comprehensive reanalysis and simulation of global weather conditions over the past several decades [52]. Therefore, we extracted the daily temperature and relative humidity data from the grids corresponding to the residential addresses of the participants. Based on the definitions of the entire pregnancy period, early pregnancy, mid-pregnancy, and late pregnancy, we calculated the average exposure levels of temperature and relative humidity for each participant.

### Statistical analysis

First, to ensure the representativeness of data, we compared the baseline information of the participants (*n* = 17,240) who were ultimately included in our study with that of the source population of the cohort (*N* = 32,176). The baseline information compared included household registration type, maternal education, occupation, maternal age, pre-pregnancy BMI, maternal birth conditions, method of conception, season of conception, gravidity, parity, alcohol consumption during pregnancy, maternal smoking, passive smoking during pregnancy, folic acid and multivitamins use before pregnancy, folic acid and multivitamins use during early pregnancy, and maternal medical status before pregnancy.

Next, statistical descriptions were provided for the exposure concentrations of PM_2.5_ and its constituents, as well as meteorological data (temperature and humidity) during each trimester and the entire pregnancy for the participants. Chi-square tests were used to examine differences in demographic characteristics between PTB and term birth.

Thirdly, we established three models to examine the relationship between PM_2.5_ with constituents exposure during each trimester and the entire pregnancy and PTB. The details of each model are summarized in Table 1.

**Table 1:** Summary of the three Generalized Additive Models (GAMs) used to analyze the relationship between PM_2.5_ and its constituents exposure and PTB

Model	Adjusted variables	Additional adjustments
Crude Model I	PM_2.5_/SO_4_^2−^/NO_3_^−^/BC/OM/NH_4_^+^	Temperature, humidity
Adjust Model II	PM_2.5_/SO_4_^2−^/NO_3_^−^/BC/OM/NH_4_^+^ + Demographic factors (household registration type, maternal education, occupation, maternal age, BMI before pregnancy, maternal birth conditions, maternal medical status before pregnancy)	Temperature, humidity
Adjust Model III	PM_2.5_/SO_4_^2−^/NO_3_^−^/BC/OM/NH_4_^+^ + Demographic factors + Pregnancy-related factors (mode of conception, season of conception, gravidity, parity, alcohol consumption during pregnancy, maternal smoking, passive smoking during pregnancy, folic acid and multivitamins before and during early pregnancy, maternal medical status during pregnancy, infant sex)	Temperature, humidity

*PM*_*2.5*_ particulate matter with an aerodynamic diameter ≤ 2.5 µm, *BC* Black carbon, *NH*_*4*_^+^ ammonium, *NO*_*3*_^*−*^ Nitrate, *OM* Organic matter, *SO*_*4*_^*2−*^ Sulfate

Fourthly, referring to previous studies [53, 54], the degrees of freedom for the cubic spline functions for temperature and humidity vary. We separately changed the degrees of freedom (*df* = 3, 4, 5) to fit the GAM. The model with the smallest Akaike Information Criterion (AIC) value was selected. The potential non-linear association between PM_2.5_ and its constituents exposure and PTB was analyzed.

Fifthly, we divided the PM_2.5_ and constituent concentrations at each trimester and the entire pregnancy into four equal parts, and categorized the exposure concentrations of PM_2.5_ and its constituents as covariates. The association between PTB and maternal exposure to PM_2.5_ and its constituents was estimated using GAM. The median of PM_2.5_ and constituent concentrations for each equal division in each trimester and the entire pregnancy was calculated as a new variable and then incorporated into the GAM to further conduct trend analysis separately.

Finally, to explore potential effect modifiers, we fitted separate models and obtained the odds ratios (ORs) and 95% confidence intervals (CIs) by maternal age, BMI before pregnancy, season of conception, maternal medical status during pregnancy, infant sex, and temperature regarding the third trimester.

### Sensitivity analysis

To examine the robustness of the results, the following sensitivity analyses were also performed. First, we conducted a sensitivity analysis by adjusting for the husband's age, level of education, type of household registration, and smoking status. Next, we removed the infants with gestational age of less than 28 weeks and the covariable of infant sex, and evaluated the association between PM_2.5_ and its constituents and PTB.

The effects of air pollution on PTB were presented as ORs and their 95% CIs associated with an interquartile range (IQR) increase in PM_2.5_ and its constituents. All statistical tests were two-sided and a *P*-value of < 0.05 was considered to be statistically significant. All statistical analyses were performed using R version 3.4.0 (https://www.r-project.org), with the packages “splines”, “Epi”, “plyr”, “lubridate”, “ggplot2”, “readxl”, “mgcv”, “car”, “psych”, “dplyr”, “VIM”, “gmodels”, “gridExtra”, “caret”, and “broom” were used.

## Results

### Characteristics of study participants

The results of the comparison between the participants included in our study and the source population of the birth cohort are shown in Supplementary Table S3. No statistically significant differences were observed in most variables between the participants in our study and the source population (*P* > 0.05), except for household registration type, maternal age, season of pregnancy, gravidity, parity, and mode of conception. Therefore, the participants we included can represent the source population of the birth cohort to some extent. Among the included 17,240 singleton live births, 1,032 (6.0%) were PTBs. The demographic characteristics and birth outcomes of the participants are presented in Table 2. The rate of PTB was higher among mothers who were over 35 years old at delivery (7.20%), had a high school education level (7.40%), had a pre-pregnancy BMI of 24 or above (7.67%), were multiparous (6.39%), were diagnosed with diabetes or hypertension before pregnancy (18.38%), were diagnosed with gestational diabetes or hypertension during pregnancy (8.02%), were born preterm themselves (9.36%), and had male infants (6.45%).

**Table 2:** Demographic characteristics of study participants

Variables	Term births (%)	Preterm births (%)	Total births		*χ* ^*2*^	*P*
			N	Percentage		
N	16208 (94.00)	1032 (6.00)	17240	100.00		
Mother						
Household registration type					0.717	0.397
Local	9849 (94.14)	613 (5.86)	10462	60.68		
Non-local	6354 (93.83)	418 (6.17)	6772	39.28		
Missing	5 (83.33)	1 (16.67)	6	0.03		
Maternal age					19.642	< 0.001***
< 30	4415 (94.91)	237 (5.09)	4652	26.98		
30–35	7343 (94.23)	450 (5.77)	7793	45.20		
> 35	4450 (92.81)	345 (7.20)	4795	27.81		
Maternal education					9.007	0.029*
Middle school or below	997 (93.97)	64 (6.03)	1061	6.15		
Senior high school	1489 (92.60)	119 (7.40)	1608	9.33		
College	11618 (94.31)	701 (5.69)	12319	71.46		
Master or above	2104 (93.43)	148 (6.57)	2252	13.06		
Occupation					8.707	0.191
Professional technicians	4136 (94.60)	236 (5.40)	4372	25.36		
Financial services	2584 (93.66)	175 (6.34)	2759	16.00		
Government official	587 (92.30)	49 (7.70)	636	3.69		
Service	1985 (93.54)	137 (6.46)	2122	12.31		
Marketing	1271 (94.71)	71 (5.29)	1342	7.78		
Media advertising	225 (94.14)	14 (5.86)	239	1.39		
Others	5420 (93.93)	350 (6.07)	5770	33.47		
BMI before pregnancy					20.422	< 0.001***
< 18.5	2547 (95.32)	1215 (4.68)	3762	21.82		
18.5–24	11409 (94.06)	720 (5.94)	12129	70.35		
≥ 24	2252 (92.33)	187 (7.67)	2439	14.15		
Maternal birth conditions						
Term birth	15522 (94.08)	976 (5.92)	16498	95.70	9.634	0.008**
Preterm birth	397 (90.64)	41 (9.36)	438	2.54		
Post-term birth	273 (95.12)	14 (4.88)	287	1.66		
Missing	16 (94.12)	1 (5.88)	17	0.10		
Mode of conception					2.650	0.104
Natural conception	14754 (94.11)	924 (5.89)	15678	90.94		
Assisted reproductive techniques	1453 (93.08)	108 (6.92)	1561	9.05		
Missing	1 (100.00)	0 (0.00)	1	0.01		
Season of conception					1.273	0.259
Cold seasons (Nov. to Apr.)	7231 (93.79)	479 (6.21)	7710	44.72		
Warm seasons (May to Oct.)	8977 (94.20)	553 (5.80)	9530	55.28		
Gravidity					1.907	0.167
1	5778 (94.35)	346 (5.65)	6124	35.52		
> 1	10430 (93.83)	686 (6.17)	11116	64.48		
Parity					4.544	0.033*
Primiparous	8501 (94.38)	506 (5.62)	9007	52.24		
Multiparious	7707 (93.61)	526 (6.39)	8233	47.76		
Maternal smoking					-	0.899
Never	15868 (94.01)	1011 (5.99)	16879	97.91		
Past	283 (94.33)	17 (5.67)	300	1.74		
Current	57 (93.44)	4 (6.56)	61	0.35		
Passive smoking during pregnancy					0.738	0.390
No	12901 (94.09)	810 (5.91)	13711	79.53		
Yes	3306 (93.71)	222 (6.29)	3528	20.46		
Missing	1 (100.00)	0 (0.00)	1	0.01		
Alcohol consumption during pregnancy					3.165	0.205
Never	6345 (93.72)	425 (6.28)	6770	39.27		
Often	607 (93.10)	45 (6.90)	652	3.78		
Seldom	9171 (94.27)	557 (5.73)	9728	56.43		
Missing	85 (94.44)	5 (5.56)	90	0.52		
Folic acid or multivitamins before pregnancy					3.105	0.212
None	5716 (93.80)	378 (6.20)	6094	35.35		
Folic acid or multivitamins	8069 (93.95)	520 (6.05)	8589	49.82		
Both	2423 (94.76)	134 (5.24)	2557	14.83		
Folic acid or multivitamins during early pregnancy					0.476	0.788
None	1080 (93.83)	71 (6.17)	1151	6.68		
Folic acid or multivitamins	11003 (93.95)	708 (6.05)	11711	67.93		
Both	4125 (94.22)	253 (5.78)	4378	25.39		
Maternal medical status before pregnancy					37.309	< 0.001***
No	16036 (94.10)	1005 (5.90)	17041	98.85		
Yes	111 (81.62)	25 (18.38)	136	0.79		
Missing	61 (96.83)	2 (3.17)	63	0.37		
Maternal medical status during pregnancy					45.466	< 0.001***
yes	4200 (91.98)	366 (8.02)	4566	26.48		
no	12008 (94.75)	666 (5.25)	12674	73.52		
Newborn						
Infant sex					7.192	0.007**
Boys	8591 (93.55)	592 (6.45)	9183	53.27		
Girls	7581 (94.53)	439 (5.47)	8020	46.52		
Missing	36 (97.30)	1 (2.70)	37	0.21		

*BMI* Body mass index

^*^ *p* < 0.05, **p < 0.01, ***p < 0.001. ‘-’:Fisher's Exact Test

### Exposure to air pollutants

The exposure concentrations of PM_2.5_ and its constituents during each trimester and the whole pregnancy, along with meteorological data, are shown in Supplementary Table S4.

The average concentrations and standard deviations (mean ± SD) of PM_2.5_ during the entire pregnancy, first trimester, second trimester, and third trimester were 20.24 ± 2.50 μg/m^3^, 20.93 ± 6.15 μg/m^3^, 20.92 ± 6.04 μg/m^3^, and 18.57 ± 5.44 μg/m^3^, respectively, all of which were below the annual average Grade 2 National Ambient Air Quality Standard (NAAQS) of 35 μg/m^3^. The average temperature and relative humidity were the highest in the third trimester, at 23.13 ℃ and 79.40%, respectively. The proportion of the five constituents of PM_2.5_ remained consistent across different pregnancy stages, with organic matter (OM) having the highest proportion, followed by sulfate (SO_4_^2−^), nitrate (NO_3_^−^), and ammonium (NH_4_^+^), while black carbon (BC) had the lowest proportion.

### Association between PTB risk and exposure to pollutants

As shown in Supplementary Table S5, S6 and Table 3, we selected adjusted model III as the final model because it produced the best model fitting results, as judged by lowest value of Akaike Information Criterion (AIC). As shown in Supplementary Table S7, we selected a cubic spline function with a degree of freedom of 5 to fit the temperature and humidity. Supplementary Figure S2-S5 show the non-linear association between PM_2.5_ and its constituents exposures and PTB in each trimester and the whole pregnancy. The results of the non-linear relationship trend between PM_2.5_ and its constituent exposure concentrations with PTB indicated that these are no significant associations in the first and second trimesters, except for the third trimester. For the third trimester, with increasing concentrations of PM_2.5_, BC, OM, and SO_4_^2−^, the risk of PTB shows an upward trend. However, the association of NH_4_^+^ and NO_3_^−^ with PTB exhibits an inverted "U"-shaped trend. With increased exposure to NO_3_^−^ and NH_4_^+^ throughout the entire pregnancy, the risk of PTB tends to rise, except for PM_2.5_, BC, OM, and SO_4_^2−^.

**Table 3:** Odds ratios (95% CI) of PTB per IQR increase in PM_2.5_ and its constituents concentrations in each trimester and the entire pregnancy, adjusted for different models (*df* = 5)

Air pollutants	Model I			Model II			Model III		
	OR (95%*CI*)	*P* Value	AIC	OR (95%*CI*)	*P* Value	AIC	OR (95%*CI*)	*P* Value	AIC
PM_2.5_									
Trimester 1	0.86 (0.70,1.05)	0.138	7815.378	0.90 (0.73,1.10)	0.312	7729.537	0.93 (0.75,1.14)	0.481	7663.254
Trimester 2	0.91 (0.74,1.12)	0.364	7812.398	0.94 (0.76,1.15)	0.536	7727.020	0.96 (0.78,1.18)	0.699	7664.086
Trimester 3	2.76 (2.14,3.57)	< 0.001***	7395.471	2.69 (2.08,3.47)	< 0.001***	7311.145	3.23 (2.49,4.19)	< 0.001***	7208.622
Entire pregnancy	1.01 (0.84,1.21)	0.894	6536.801	1.04 (0.87,1.25)	0.672	6501.431	1.12 (0.92,1.35)	0.255	6412.878
BC									
Trimester 1	0.85 (0.71,1.01)	0.069	7814.239	0.88 (0.74,1.06)	0.175	7728.712	0.90 (0.75,1.08)	0.250	7662.408
Trimester 2	0.90 (0.75,1.08)	0.253	7811.879	0.93 (0.77,1.11)	0.404	7726.688	0.95 (0.79,1.14)	0.566	7663.895
Trimester 3	2.40 (1.90,3.04)	< 0.001***	7402.209	2.34 (1.86,2.96)	< 0.001***	7317.822	2.71 (2.14,3.43)	< 0.001***	7218.063
Entire pregnancy	0.87 (0.73,1.03)	0.112	6534.311	0.89 (0.75,1.07)	0.208	6500.044	0.97 (0.81,1.16)	0.720	6414.099
NH_4_^+^									
Trimester 1	0.97 (0.68,1.39)	0.866	7817.362	1.05 (0.73,1.52)	0.800	7730.309	1.16 (0.79,1.69)	0.455	7663.045
Trimester 2	0.94 (0.65,1.34)	0.728	7812.930	0.95 (0.66,1.35)	0.766	7727.150	0.98 (0.69,1.40)	0.912	7664.114
Trimester 3	1.62 (1.20,2.20)	0.002**	7444.758	1.55 (1.15,2.10)	0.004**	7359.374	1.75 (1.29,2.38)	< 0.001***	7271.407
Entire pregnancy	0.87 (0.69,1.11)	0.275	6535.553	0.89 (0.70,1.14)	0.367	6500.749	0.99 (0.77,1.27)	0.946	6414.212
NO_3_^−^									
Trimester 1	0.79 (0.57,1.10)	0.164	7816.406	0.84 (0.60,1.16)	0.285	7729.902	1.01 (0.69,1.47)	0.975	7663.692
Trimester 2	0.94 (0.67,1.32)	0.725	7812.876	0.96 (0.69,1.35)	0.825	7727.159	1.00 (0.71,1.40)	0.990	7664.114
Trimester 3	1.36 (1.02,1.82)	0.039*	7450.052	1.32 (0.99,1.76)	0.062	7363.894	1.42 (1.06,1.89)	0.019*	7278.624
Entire pregnancy	1.15 (0.89,1.50)	0.285	6535.778	1.19 (0.92,1.55)	0.184	6499.986	1.34 (1.02,1.75)	0.032*	6409.861
OM									
Trimester 1	0.84 (0.69,1.02)	0.084	7814.584	0.88 (0.73,1.07)	0.206	7728.963	0.90 (0.74,1.10)	0.301	7662.682
Trimester 2	0.92 (0.76,1.11)	0.392	7812.464	0.95 (0.78,1.15)	0.574	7727.074	0.97 (0.80,1.18)	0.751	7664.127
Trimester 3	2.54 (1.98,3.26)	< 0.001***	7401.793	2.48 (1.94,3.19)	< 0.001***	7317.350	2.98 (2.31,3.83)	< 0.001***	7215.114
Entire pregnancy	0.92 (0.76,1.10)	0.337	6535.904	0.94 (0.79,1.13)	0.528	6501.229	1.01 (0.84,1.22)	0.898	6414.200
SO_4_^2−^									
Trimester 1	0.87 (0.73,1.04)	0.118	7815.068	0.90 (0.76,1.07)	0.250	7729.190	0.91 (0.76,1.09)	0.325	7662.762
Trimester 2	0.94 (0.78,1.12)	0.474	7812.700	0.96 (0.80,1.15)	0.650	7727.196	0.98 (0.82,1.18)	0.836	7664.190
Trimester 3	2.61 (2.07,3.29)	< 0.001***	7391.233	2.53 (2.01,3.19)	< 0.001***	7307.811	3.03 (2.39,3.83)	< 0.001***	7202.829
Entire pregnancy	0.74 (0.61,0.89)	0.002**	6527.085	0.77 (0.63,0.92)	0.006**	6493.940	0.84 (0.69,1.02)	0.084	6411.293

*PM*_*2.5*_ particulate matter with an aerodynamic diameter ≤ 2.5 µm, *BC* Black carbon, *NH*_*4*_^+^ ammonium, *NO*_*3*_^*−*^ Nitrate, *OM* Organic matter, *SO*_*4*_^*2−*^ Sulfate

Model I, a crude model, just includes the concentration of PM2.5 or its constituents. Model II additionally adjusts for demographic information, including household registration type, maternal education, occupation, maternal age, BMI before pregnancy, maternal birth conditions, and maternal medical status before pregnancy. Model III further adjusts for mode of conception, season of conception, gravidity, parity, alcohol consumption during pregnancy, maternal smoking, passive smoking during pregnancy, folic acid and multivitamins before pregnancy, folic acid and multivitamins during early pregnancy, maternal medical status during pregnancy, and infant sex. The above models were all adjusted for temperature and humidity

^*^p < 0.05, **p < 0.01, ***p < 0.001

The results of the relationship between exposure to PM_2.5_ and its constituents during each trimester and the entire pregnancy with PTB are shown in Table 3. We observed that exposure to PM_2.5_ and its constituents in the third trimester was associated with PTB, but not for exposure in the first or second trimester. With each increase of IQR increase in PM_2.5_, the risk of PTB increased by 2.23 times. The ORs of PTB for each IQR increase in the concentrations of BC, NH_4_^+^, NO_3_^−^, OM, and SO_4_^2−^ were greater than 1, with a significant difference. Constituent analysis revealed that exposure to SO_4_^2−^ (OR: 3.03, 95% CI: 2.39–3.83) and OM (OR: 2.98, 95% CI: 2.31–3.83) had effects on PTB comparable to those of PM_2.5_ (OR: 3.23, 95% CI: 2.49–4.19). The relationship between PM_2.5_ and PTB over the entire pregnancy suggests that PM_2.5_ is a risk factor (OR = 1.12), but the 95% CI includes 1, indicating no statistically significant association. However, the analysis results of PM_2.5_ constituents show that exposure to NO_3_^−^ is associated with PTB (OR: 1.34, 95% CI: 1.02–1.75).

The trend analysis results of PM_2.5_ and its constituents exposures during each trimester and the entire pregnancy are shown in Table 4. The results of the relationship between PM_2.5_ exposure in the third trimester and PTB suggest that, compared to the first quartile (Q1), the ORs for PTB increase with each quartile of PM_2.5_ concentration: 1.84 for the second quartile (Q2), 3.35 for the third quartile (Q3), and 4.63 for the fourth quartile (Q4), with a *P* for trend value of < 0.001. BC, NH_4_^+^, NO_3_^−^, OM, and SO_4_^2−^ exhibit an upward trend in the risk of PTB with increasing levels of exposure concentration (all *P* for trend values < 0.05). As the exposure concentration level of PM_2.5_ increases throughout the entire pregnancy, there is no upward trend in the OR for PTB (*P* for trend value = 0.885). The constituent analysis results indicate that, compared to the first quartile (Q1), the risk of PTB also increases with the rising exposure concentrations of NH_4_^+^ and NO_3_^−^ (*P* for trend value < 0.05). Conversely, exposure to SO_4_^2−^ shows a decreasing trend in the risk of PTB with increasing concentration (*P* for trend value = 0.019).

**Table 4:** Exposure to PM_2.5_ and its constituents and the associated odds ratios of PTB

Trimester 1			Trimester 2			Trimester 3			The entire pregnancy		
Pollutants (μg/m^3^) (median [range])	*OR (95% CI)*	*P* for trend	Pollutants (μg/m^3^) (median [range])	*OR (95% CI)*	*P* for trend	Pollutants (μg/m^3^) (median [range])	*OR (95% CI)*	*P* for trend	Pollutants (μg/m^3^) (median [range])	*OR (95% CI)*	*P* for trend
PM_2.5_			PM_2.5_			PM_2.5_			PM_2.5_		
Q1 (13.27 [< 16.20])	Reference		Q1 (13.18 [< 16.14])	Reference		Q1 (11.69 [< 13.76])	Reference		Q1 (17.17 [< 18.30])	Reference	
Q2 (18.71 [16.20,21.01])	1.28 (0.98, 1.67)		Q2 (18.91 [16.14,21.27])	0.98 (0.78, 1.22)		Q2 (16.38 [13.76,18.81])	1.84 (1.40, 2.42)		Q2 (19.34 [18.30, 20.36])	0.76 (0.59, 0.98)	
Q3 (23.04 [21.01,25.03])	1.31 (0.91, 1.9)		Q3 (23.12 [21.27,24.94])	0.91 (0.66, 1.25)		Q3 (20.65 [18.81,22.88])	3.35 (2.34, 4.79)		Q3 (21.27 [20.36, 22.11])	0.81 (0.58, 1.13)	
Q4 (28.36 [> 25.03])	1.04 (0.69, 1.57)	0.420	Q4 (27.99 [> 24.94])	0.85 (0.58, 1.24)	0.388	Q4 (25.04 [> 22.88])	4.63 (3.01, 7.13)	< 0.001***	Q4 (23.19 [> 22.11)	0.96 (0.68, 1.37)	0.885
Trimester 1			Trimester 2			Trimester 3			The entire pregnancy		
Pollutants (μg/m^3^) (median [range])	*OR (95% CI)*	*P* for trend	Pollutants (μg/m^3^) (median [range])	*OR (95% CI)*	*P* for trend	Pollutants (μg/m^3^) (median [range])	*OR (95% CI)*	*P* for trend	Pollutants (μg/m^3^) (median [range])	*OR (95% CI)*	*P* for trend
BC			BC			BC			BC		
Q1 (0.74 [< 0.92])	Reference		Q1 (0.74 [< 0.91])	Reference		(0.65 [< 0.77])	Reference		Q1 (0.97 [< 1.04])	Reference	
Q2 (1.07 [0.92,1.19])	1.06 (0.81, 1.38)		Q2 (1.08 [0.91,1.20])	1.24 (0.91, 1.69)		(0.92 [0.77,1.06])	2.33 (1.76, 3.08)		Q2 (1.10 [1.04,1.15])	0.76 (0.59, 0.98)	
Q3 (1.30 [1.19,1.43])	1.07 (0.75, 1.53)		Q3 (1.30 [1.20,1.42])	1.02 (0.69, 1.50)		(1.16 [1.06,1.28])	4.24 (2.87, 6.26)		Q3 (1.21 [1.15,1.27])	0.88 (0.64, 1.21)	
Q4 (1.68 [> 1.43])	0.87 (0.59, 1.3)	0.130	Q4 (1.64 [> 1.42])	0.94 (0.62, 1.42)	0.340	(1.41 [> 1.28])	5.47 (3.49, 8.56)	< 0.001***	Q4 (1.33 [> 1.27])	0.81 (0.58, 1.13)	0.276
NH_4_^+^			NH_4_^+^			NH_4_^+^			NH_4_^+^		
Q1 (0.95 [< 1.16])	Reference		Q1 (0.95 [< 1.17])	Reference		Q1 (0.83 [< 0.99])	Reference		Q1 (1.32 [< 1.49])	Reference	
Q2 (1.38 [1.16,1.60])	1.14 (0.86, 1.52)		Q2 (1.41 [1.17,1.63])	1.27 (0.93, 1.74)		Q2 (1.18 [0.99,1.38])	2.80 (2.08, 3.77)		Q2 (1.63 [1.49,1.72])	1.04 (0.70, 1.54)	
Q3 (1.95 [1.60,2.30])	1.43 (0.93, 2.2)		Q3 (1.98 [1.63,2.32])	1.02 (0.65, 1.59)		Q3 (1.54 [1.38,1.85])	7.07 (4.61, 10.85)		Q3 (1.78 [1.72,1.84])	1.04 (0.63, 1.72)	
Q4 (2.66 [> 2.30])	1.33 (0.78, 2.28)	0.481	Q4 (2.66 [> 2.32])	0.94 (0.55, 1.58)	0.689	Q4 (2.38 [> 1.85])	4.12 (2.38, 7.12)	0.013 *	Q4 (1.92 [> 1.84])	1.54 (0.91, 2.58)	0.022 *
NO_3_^−^			NO_3_^−^			NO_3_^−^			NO_3_^−^		
Q1 (1.13 [< 1.43])	Reference		Q1 (1.16 [< 1.47])	Reference		Q1 (1.02 [< 1.23])	Reference		Q1 (1.70 [< 2.01])	Reference	
Q2 (1.38 [1.43,2.09])	1.14 (0.86, 1.52)		Q2 (1.82 [1.47,2.14])	1.08 (0.86, 1.36)		Q2 (1.49 [1.23,1.77])	2.22 (1.70, 2.91)		Q2 (2.24 [2.01,2.39])	1.57 (1.03, 2.39)	
Q3 (2.71 [2.09,3.34])	1.26 (0.82, 1.95)		Q3 (2.73 [2.14,3.37])	1.03 (0.71, 1.51)		Q3 (2.02 [1.77,2.44])	5.08 (3.43, 7.53)		Q3 (2.48 [2.39,2.57])	1.91 (1.12, 3.25)	
Q4 (3.99 [> 3.34])	0.94 (0.56, 1.58)	0.723	Q4 (3.94 [> 3.37])	0.99 (0.61, 1.62)	0.843	Q4 (3.42 [> 2.44])	3.12 (1.82, 5.37)	0.019 *	Q4 (2.68 [> 2.57])	2.71 (1.56, 4.70)	< 0.001***
OM			OM			OM			OM		
Q1 (3.60 [< 4.52])	Reference		Q1 (3.61 [< 4.53])	Reference		Q1 (3.26 [< 3.83])	Reference		Q1(4.84 [< 5.18])	Reference	
Q2 (5.31 [4.52,5.97])	1.2 (0.91, 1.59)		Q2 (5.36 [4.53,6.04])	0.97 (0.78,1.22)		Q2 (4.56 [3.83,5.29])	2.00 (1.54, 2.60)		Q2(5.47 [5.18,5.78])	0.77 (0.60, 1.00)	
Q3 (6.55 [5.97,7.15])	1.3 (0.89, 1.89)		Q3 (6.54 [6.04,7.10])	0.89 (0.65,1.22)		Q3 (5.82 [5.29,6.48])	3.58 (2.49, 5.15)		Q3(6.06 [5.78,6.30])	0.70 (0.50, 0.97)	
Q4 (8.23 [> 7.15])	1.04 (0.69, 1.58)	0.376	Q4 (8.10 [> 7.10])	0.85 (0.58,1.24)	0.391	Q4 (7.13 [> 6.48])	5.36 (3.48, 8.25)	< 0.001***	Q4(6.60 [> 6.30])	0.80 (0.56, 1.13)	0.352
Trimester 1			Trimester 2			Trimester 3			The entire pregnancy		
Pollutants (μg/m^3^) (median [range])	*OR (95% CI)*	*P* for trend	Pollutants (μg/m^3^) (median [range])	*OR (95% CI)*	*P* for trend	Pollutants (μg/m^3^) (median [range])	*OR (95% CI)*	*P* for trend	Pollutants (μg/m^3^) (median [range])	*OR (95% CI)*	*P* for trend
SO_4_^2−^			SO_4_^2−^			SO_4_^2−^			SO_4_^2−^		
Q1 (2.53 [< 3.21])	Reference		Q1 (2.51 [< 3.17])	Reference		Q1 (2.22 [< 2.65])	Reference		Q1 (3.40 [< 3.61])	Reference	
Q2 (3.77 [3.21,4.18])	1.2 (0.91, 1.58)		Q2 (3.80 [3.17,4.22])	1.22 (0.90, 1.67)		Q2 (3.23 [2.65,3.76])	2.36 (1.79, 3.12)		Q2 (3.79 [3.61,4.00])	0.73 (0.57, 0.94)	
Q3 (4.50 [4.18,4.94])	1.28 (0.89, 1.84)		Q3 (4.52 [4.22,4.94])	1.21 (0.82, 1.77)		Q3 (4.10 [3.76,4.47])	3.85 (2.61, 5.69)		Q3 (4.21 [4.00,4.39])	0.71 (0.51, 0.99)	
Q4 (5.76 [> 4.94])	1.01 (0.69, 1.5)	0.238	Q4 (5.60 [> 4.94])	1.15 (0.76, 1.72)	0.910	Q4 (4.94 [> 4.47])	5.00 (3.21, 7.78)	< 0.001***	Q4 (4.59 [> 4.39])	0.64 (0.45, 0.92)	0.019 *

*PM*_*2.5*_ particulate matter with an aerodynamic diameter ≤ 2.5 µm, *BC* Black carbon, *NH*_*4*_^+^ ammonium, *NO*_*3*_^*−*^ Nitrate, *OM* Organic matter, *SO*_*4*_^*2−*^ Sulfate, *OR* odds ratios, *CI* confidence interval

^*^p < 0.05; **p < 0.01; ***p < 0.001. The model was adjusted for household registration type, maternal age, BMI before pregnancy, maternal education, occupation, maternal birth conditions, maternal medical status before pregnancy, season of conception, gravidity, parity, mode of conception, maternal smoking, alcohol consumption during pregnancy, passive smoking during pregnancy, folic acid and multivitamins before pregnancy, folic acid and multivitamins during early pregnancy, maternal medical status during pregnancy, infant sex, temperature and relative humidity

### Stratified analyses

**Fig. 2:**
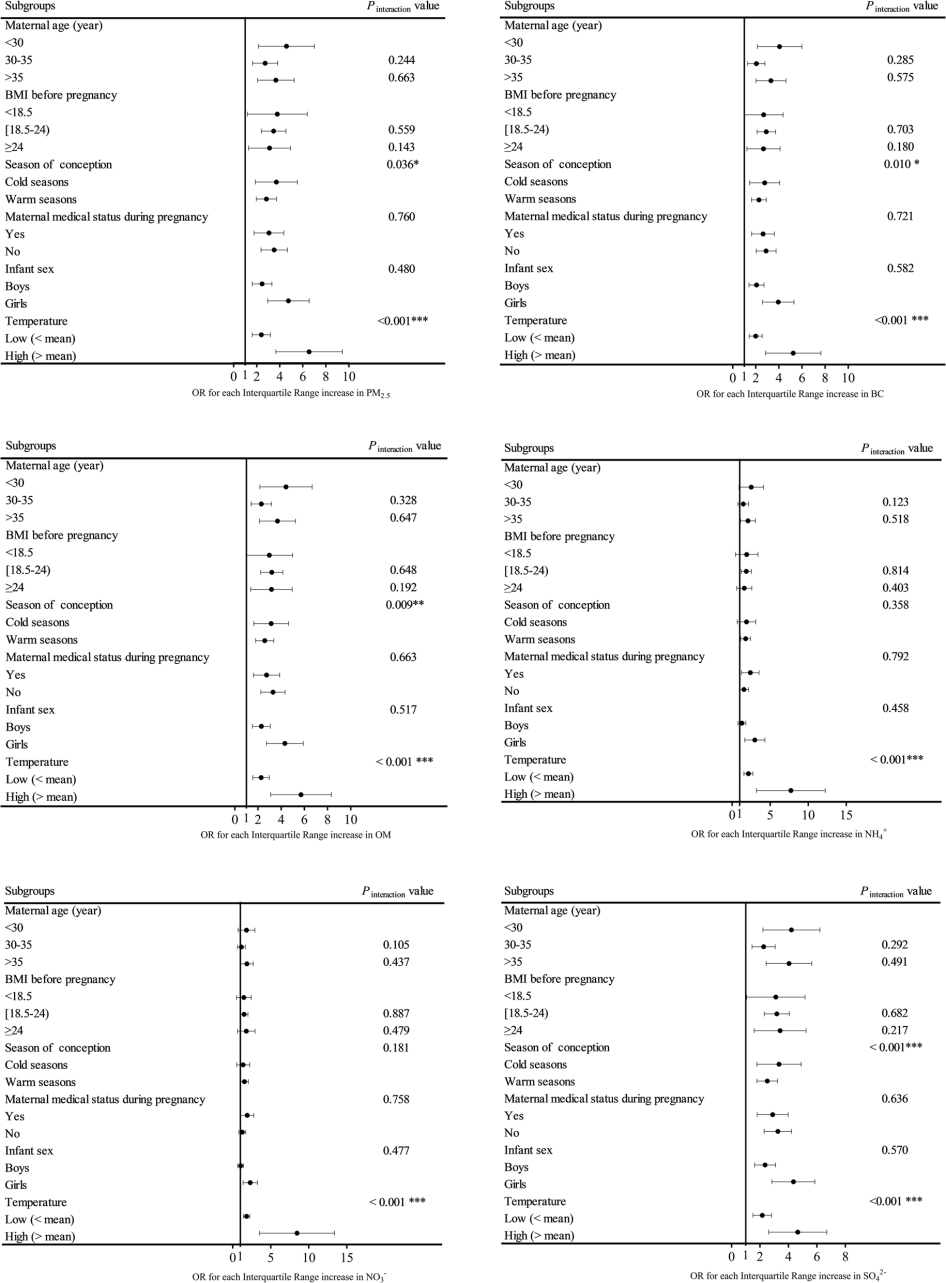
shows the associations between PM_2.5_ and its constituents exposures during the third gestational period and PTB stratified by maternal age, BMI before pregnancy, season of conception, maternal medical status during pregnancy, infant sex and temperature. Abbreviations: PM_2.5_, particulate matter with an aerodynamic diameter ≤ 2.5 µm; BC, Black carbon; NH_4_^+^, ammonium; NO_3_^−^, Nitrate; OM, Organic matter; SO_4_^2−^: Sulfate

Figure 2 presents the associations between PM_2.5_ and its constituents exposures during the third gestational period and PTB, stratified by maternal age, BMI before pregnancy, season of conception, maternal medical status during pregnancy, infant sex and temperature. Pregnant women who conceived during the cold season (November to April) are more likely to be sensitive to PM_2.5_ and its constituents, resulting in a higher risk of PTB (*P*-value for interaction < 0.05), except for NH_4_^+^ (*P*-value for interaction = 0.3578) and NO_3_^−^ (*P*-value for interaction = 0.1810). Additionally, we found that women exposed to higher temperatures (> mean temperature) during pregnancy have a higher risk of PTB (all *P-*values for interaction < 0.001).

### Sensitivity analysis

First, sensitivity analysis in the main model was further adjusted for the father's educational level, type of household registration, age, and smoking status and the results are presented in Supplementary Table S8, which are consistent with the main findings. Next, after excluding infants with a gestational age of less than 28 weeks and removing the covariate of infant sex, sensitivity analysis was conducted, with the results presented in Supplementary Table S9. The results of the relationship between exposure to PM_2.5_ and its constituents in third trimester and PTB are also consistent with the main findings, but there is no association between exposure to NO_3_^−^ in the entire pregnancy and PTB. Additionally, the results indicate that exposure to SO_4_^2−^ throughout the entire pregnancy is a protective factor for PTB. Overall, the sensitivity analysis suggests that our research findings are relatively robust.

## Discussion

In the context of the new environmental pollution challenges in China, based on a prospective birth cohort database, we employed a retrospective cohort study to investigate the association between exposure to low concentrations of PM_2.5_ and its constituents with PTB, and further explored potential factors through stratified analysis. We identified third trimester of pregnancy as the critical exposure window. The exposure effects of SO_4_^2−^, OM, and BC are equivalent to the total exposure of PM_2.5_. The results of the trend analysis show that exposure to PM_2.5_ and its constituents during the third trimester significantly increases the risk of PTB. The results for constituents throughout the entire pregnancy show that the risk of PTB increases with rising exposure concentrations of NO_3_^−^. A higher risk of PTB was observed among mothers who conceived during the cold season (November to April). Additionally, stronger effects of air pollutants on PTB were observed for exposure to higher temperatures compared to lower temperatures.

Numerous studies in China have investigated the association between PM_2.5_ exposure and PTB, but most were conducted under relatively high PM_2.5_ concentration levels. For instance, a study conducted in Wuhan, China, demonstrated that prenatal PM_2.5_ exposure increases the risk of PTB. In this study, the average PM_2.5_ exposure concentration during the entire pregnancy was 84.54 μg/m^3^ [55], significantly exceeding the national annual secondary concentration limit of 35 µg/m^3^. A nationwide study covering 336 cities in China reported an average PM_2.5_ exposure concentration exceeding 50 μg/m^3^ during the entire pregnancy period, with a range of 23–91 µg/m^3^ [18], which is higher than the average exposure concentration in our study (20.24 µg/m^3^). Another study conducted in Guangzhou, a major city in China with comparable economic status, geographical location, and climate to our study region, reported a weekly average PM_2.5_ exposure concentration of 32.07 μg/m^3^ [54], also higher than our average exposure level. Therefore, although our exposure concentrations remain relatively high compared to those in other developed countries, the PM_2.5_ exposure levels among the participants in this research are considered low (below the national annual secondary concentration limit of 35 μg/m^3^) when referenced against previous epidemiological studies in China. These findings provide valuable evidence on the association between air pollution and PTB under improved environmental conditions. Furthermore, the analysis of the associations between the five PM_2.5_ constituents and PTB offers critical insights for developing targeted air pollution control strategies and safeguarding maternal and infant health in the future.

Previous studies have found differing sensitive windows for PM_2.5_ exposure and PTB. A cohort study from Australia identified that the second trimester might be the critical window [53]. The strongest associations were observed in the second and third trimesters in California [56]. However, our study found that the third trimester is the critical exposure window, which contrasts with the aforementioned research findings. This discrepancy could be attributed to the different sources of PM_2.5_ exposure. The sources of PM_2.5_ in Australia and California are primarily wildfire-related PM_2.5_, which is a primary emission of wildfire smoke, whereas in our country, the main sources of PM_2.5_ are chemical fuel combustion, industrial activities, and vehicular exhaust emissions. Different sources of exposure lead to different health effects. Previous studies suggest that wildfire-related PM_2.5_ is more harmful to human health than ambient PM_2.5_ [57, 58]. Additionally, the exposure concentrations vary. Although the concentrations of PM_2.5_ in our study are relatively low within China, they are still higher compared to those in developed countries. Moreover, the exposed populations differ. The effects of factors such as race, socioeconomic status, age, and dietary habits on the risk of PTB vary. A nationwide cohort study from China found that the late stage of pregnancy as the critical exposure window [15], which is consistent with our study. We hypothesize that PTB may be the result of chronic inflammation induced by long-term exposure to PM_2.5_. Interestingly, although our sensitive window periods are consistent, the median exposure concentration of PM_2.5_ (18.81 μg/m^3^) is significantly lower than reported in the aforementioned study (48.25 μg/m^3^), which may reflect differences in the toxic effects of PM_2.5_. Previous studies have confirmed that exposure to air pollution during pregnancy can induce systemic inflammatory responses [59]. Furthermore, regardless of the concentration of air pollution, besides inflammation, it can also trigger systemic oxidative stress [60]. Oxidative stress can activate inflammatory signaling pathways, promoting the recruitment of inflammatory cells and the release of inflammatory mediators. Under the combined effects of inflammatory responses and oxidative stress, the inflammatory reactions are continuously exacerbated in the body, ultimately leading to PTB. However, some earlier studies with PM_2.5_ concentrations below 35 μg/m^3^ showed no association between PM_2.5_ exposure and PTB, and some even reported a negative correlation [29, 30, 61], which is inconsistent with our study. This discrepancy may be attributed to various factors, such as geographical differences, racial variations, and differences in the concentration and composition of PM_2.5_. Further research is needed to investigate the association between low-concentration air pollution exposure and PTB, as well as the underlying biological mechanisms.

The composition of PM_2.5_ is a key factor influencing the risk of PTB [17, 33]. PM_2.5_ composition is complex, consisting of OM, BC, SO_4_^2−^, NO_3_^−^, and NH_4_^+^. In different regions, influenced by industrial production and economic development, the proportions and sources of chemical constituents in PM_2.5_ vary, which may also result in differing health effects on pregnant women. A cohort study from Fujian, China, found that NH_4_^+^ is the main constituent of PM_2.5_ associated with PTB [62]. A study from Italy found that secondary SO_4_^2−^ and OM are associated with a higher risk of PTB compared to the total mass of PM_2.5_ [63]. However, a nationwide birth cohort study conducted in 336 cities in China found that the association between carbon constituents (organic carbon (OC) and BC) and PTB was more significant, with a 9% increase in PTB risk for each IQR increase in carbon constituent concentration [18]. Our study found that SO_4_^2−^, OM, and BC have the greatest estimated impact on PTB. For each IQR increase in concentration, the risk of PTB increased by 2.03-fold, 1.98-fold, and 1.71-fold, respectively. This may be attributed to the differential inflammatory response effects induced by different constituents. Previous studies have shown that exposure to PM_2.5_ can trigger systemic inflammation regardless of duration, with sulfate constituents playing a significant role [64]. Our results indicate that the concentration of SO_4_^2−^ ranks second after OM, suggesting that enhanced emission management of SO_4_^2−^ is necessary. Notably, BC accounts for the smallest fraction of PM_2.5_'s total mass in our results, yet its health effects are not the least significant. This may be associated with the physicochemical properties of BC. Research indicates that the BC constituent has greater toxicity or a higher oxidative potential, leading to more pronounced health effects [65]. Moreover, Eva Bongaerts et al. have found that carbon-containing air pollution particles inhaled by pregnant women can cross the placenta and accumulate there [66]. On one hand, they can induce placental inflammatory responses, and on the other hand, they can maintain the placenta in an inflammatory state for a prolonged period [67], thereby further increasing the risk of PTB. BC primarily originates from industrial emissions, the combustion of diesel and gasoline fuels, and motor vehicle emissions, while SO_4_^2−^, NH_4_^+^, and NO_3_^−^ are mainly produced through the photochemical transformation of precursor pollutants, primarily derived from fossil fuels, biofuels, coal-fired power plants, and automobile exhausts [23, 68]. The primary sources of PM_2.5_ in South China are secondary sources and traffic-related emissions [69]. Policymakers should consider the current state of environmental pollution and formulate environmental management strategies to reduce emissions of SO_4_^2−^ and BC, thereby mitigating the adverse effects of PM_2.5_ on PTB.

Temperature is an important modifying factor for adverse pregnancy outcomes, but different studies have reported varying health effects. A study in Australia found that under low-concentration air pollution conditions, exposure to moderate and low temperatures has a greater impact on PTB and low birth weight [31]. However, our study found that under low concentrations of PM_2.5_, exposure to higher temperatures has a greater impact on PTB compared to lower temperatures. This may be attributed to the combined influence of geographical differences, varying pollutant exposures, and other factors. The potential biological mechanism may be that heat exposure exacerbates the inflammatory response, thereby increasing the risk of PTB. Heat exposure, on the one hand, can directly increase oxidative stress and promote inflammatory responses, and on the other hand, it can increase the release of oxytocin, prostaglandins, and inflammatory markers. Furthermore, it can upregulate the expression of heat shock proteins, triggering inflammatory responses through various pathways [70, 71], which contribute to the development of PTB. Reduced placental blood flow may also be another important mechanism underlying PTB. Under conditions of heat exposure, to maintain normal body temperature, the thermoregulatory center redirects more blood to the body's surface, thereby increasing heat dissipation through the skin and indirectly reducing placental blood flow. Sweating is another mechanism for heat dissipation. As sweating increases, blood circulation decreases, and placental blood perfusion further declines [71], ultimately impairing fetal growth and development. Currently, the impact of temperature on adverse pregnancy outcomes is not fully understood, and variations in temperature exposure assessment and definition may be critical contributing factors. Systematic reviews and meta-analyses have found that exposure to high temperatures during pregnancy is associated with adverse pregnancy outcomes, including PTB and low birth weight (LBW) [72, 73]. However, another systematic review and meta-analysis found that exposure to extreme ambient temperatures, particularly during the third trimester of pregnancy, is associated with adverse birth outcomes, such as PTB and stillbirth [74]. Overall, due to the prolonged duration of pregnancy and the complex interplay of exposure factors during this period, it is difficult to identify specific high-risk factors for adverse birth outcomes. Future research should focus on standardizing heat exposure definitions, establishing unified exposure assessment criteria, and conducting multicenter studies to explore the association between temperature and adverse pregnancy outcomes, as well as their underlying biological mechanisms.

Significant seasonality in conception was also observed. Multiple studies have shown that pregnant women who conceive during the cold season are more likely to have an increased risk of PTB [53, 75], which aligns with our findings. Cold stimulation can increase blood viscosity and constrict peripheral blood vessels, potentially affecting placental blood perfusion and ultimately impacting the growth and development of the fetus [76–78]. Insufficient vitamin D synthesis may be a potential cause for the increased risk of adverse pregnancy outcomes among pregnant women who conceive in the cold season. Studies have shown that vitamin D deficiency not only reduces fertility [79, 80] but also increases the risk of adverse pregnancy outcomes, including PTB and miscarriage [81–83]. Vitamin D deficiency is associated with elevated levels of circulating inflammatory proteins, which may regulate the levels of C-reactive protein, thereby enhancing the inflammatory response in the body [84]. Furthermore, a deficiency in vitamin D can reduce the average concentration of estradiol [85]. Estradiol, as the predominant active form of estrogen, plays a crucial role in maintaining normal pregnancy. The potential factors and mechanisms through which the conception season may affect gestational physiology are still unclear, and further research is warranted.

Although the effects of ambient air pollution on birth outcomes have been previously investigated in China, our study differs from previous ones in terms of the study population, design, sample size, questionnaires, and PM_2.5_ exposure concentrations. First, to our knowledge, given the current state of environmental pollution in China, there are relatively few areas where the PM_2.5_ concentration is below 35 μg/m^3^. Therefore, the conditions for conducting research on the association between low concentrations of PM_2.5_ exposure and PTB are somewhat limited. This study can provide evidence for the precise implementation of environmental governance and the protection of maternal and infant health under the current circumstances of environmental pollution in China. Second, due to the limitations of economic factors and instrumentation, there is a scarcity of data on the composition of PM_2.5_. This study, by integrating PM_2.5_ composition to explore the relationship between PM_2.5_ exposure and PTB, can reflect the contribution of different constituents to the risk of PTB, providing evidence for targeted environmental governance. Third, our study is based on a prospective birth cohort, with accurate and reliable collection of demographic information from pregnant women, including their medical conditions before and during pregnancy, as well as their dietary habits during pregnancy, which enhances the robustness of the study.

Our study also has some limitations. First, as a single-center study, it has a relatively narrow range of air pollution exposure concentrations, which limits the assessment of the dose–response relationship between exposure and outcomes. Second, the assessment of air pollution exposure is not sufficiently precise. Indoor air pollution is an important contributing factor to many adverse health outcomes [86, 87], but due to a lack of data on the travel patterns of pregnant women, we cannot accurately assess the actual PM_2.5_ and constituent exposure levels for each participant. Third, the composition of polluted air is complex, including PM_2.5_, CO, O_3_, NO_2_, and SO_2_, among others. Different gaseous pollutants can interact with each other. Our study only assessed the association between PM_2.5_ and its constituents and PTB without accounting for the health effects of other pollutants. As a result, the association between PM_2.5_ and its constituents' exposure and PTB may be underestimated. Fourth, the participants included in our study were all live births. Some fetuses that would have been born preterm may have been particularly sensitive to air pollution due to individual susceptibility, ultimately not surviving until birth, which introduces uncertainty into the study results.

## Conclusions

Our study found that exposure to low concentrations of PM_2.5_ can increase the risk of PTB. Exposure to SO_4_^2−^, OM, and BC has effects comparable to the total effect of PM_2.5_ exposure. The third trimester of pregnancy might be the critical susceptibility window. Stratified analysis found that pregnant women exposed to higher temperatures and those who are pregnant during the cold season may be more susceptible to PTB. Currently, although significant progress has been made in air pollution prevention and control in China, our results indicate that exposure to low concentrations of PM_2.5_ still increases the risk of PTB. Different constituents of PM_2.5_ have varying effects on PTB due to differences in their concentrations and physicochemical properties, highlighting the need to properly characterize the sources contributing to PM_2.5_ and underscoring the importance of further strengthening environmental governance to reduce PM_2.5_ concentrations.

## Supplementary Information

Supplementary Material 1.

## Data Availability

Data will be made available on reasonable request. All inquiries about data should be sent to fanfan20221125@163.com. The person responsible for handling inquiries is Ms. Jingjie Fan from the Department of Preventive Healthcare, Shenzhen Maternity and Child Healthcare Hospital, Southern Medical University.
