# Supplementary material for: Exposure to low concentrations of PM2.5 and its constituents with preterm birth in Shenzhen, China: a retrospective cohort study
Source: BMC Public Health. 2025 Apr 7;25:1295. doi: 10.1186/s12889-025-22489-7 (PMC11974226; doi:10.1186/s12889-025-22489-7)
Supplement: Supplementary file 1 — Supplementary Material 1. [file 12889_2025_22489_MOESM1_ESM.docx]

**Supplementary material**

Exposure to low concentrations of PM_2.5_ and its constituents with preterm birth: a retrospective cohort study in Shenzhen, China

**Contents**

**Text S1** and corresponding **Table S1.**

**Text S2** and corresponding **Table S2**.

**Text S3** and corresponding **Figure S1**.

**Text S4** and corresponding **Table S3**.

**Text S5** and corresponding **Table S4**.

**Text S6** and corresponding **Table S5 and Table S6**.

**Text S7** and corresponding **Table S7**.

**Text S8** and corresponding **Figure S2-5**.

**Text S9** and corresponding **Table S8 and Table S9**.

**Supplementary text**

**Text S1.** **Table S1** shows the questionnaire information.

**Text S2.** **Table S2** provides information on birth outcomes.

**Text S3.** **Figure S1** shows the potential confounders using a Directed Acyclic Graph (DAG).

**Text S4.** **Table S3** shows the results of the comparison between the participants included in our study and the source population of the birth cohort.

**Text S5. Table S4** shows the exposure concentrations of PM_2.5_ and its components during each trimester and the entire pregnancy, along with meteorological data.

**Text S6.** **Table S5 and Table S6** show the odds ratios (95% CI) of PTB per IQR increase in PM_2.5_ and its constituents concentrations in each trimester and the entire pregnancy, across different degrees of freedom and models.

**Text S7.** **Table S7** shows the results of the AIC for cubic splines with different degrees of freedom in Model III at different stages of pregnancy.

**Text S8. Figure S2-5** show the non-linear association between PM_2.5_ exposure and its constituents with PTB in each trimester and the whole pregnancy.

**Text S9.** **Table S8 and Table S9** show the results of sensitivity analysis, with adjusted models.

**Table S1 The questionnaire information**

| Type of variable | Classification declaration |
| --- | --- |
| Demographic characteristics | |
| Home address | province city district street number |
| Maternal household registration type | □Local  □Non-local |
| Maternal age (years) | Years  (Categorized as three classes: <30 years, 30-35 years, >35 years) |
| Maternal education | □Middle school or below  □Senior high school  □College  □Master or above |
| Maternal occupation | □Professional Technicians  □Financial Services  □Government official  □Service  □Marketing  □Media Advertising  □Others |
| Maternal height (cm) | cm |
| Maternal weight before pregnancy (kg) | kg |
| Body mass index (BMI) before pregnancy | BMI before pregnancy was calculated as weight in kilograms divided by height in meters squared (kg/m^2^) (Categorized as three classes: <18.5, [18.5-24), [24-28), ≥28) |
| Maternal Birth Conditions | □Preterm birth  □Term birth  □Post-term birth |
| Paternal household registration type | □Local  □Non-local |
| Paternal age (years) | Years  (Categorized as three classes: <30 years, 30-35 years, >35 years) |
| Paternal education | □Middle school or below  □Senior high school  □College  □Master or above |
| Pregnancy conditions | |
| Last menstrual period | Date: |
| Mode of conception | □Natural conception  □Assisted Reproductive Techniques |

[continued]

Table S1 [continued]

| Type of variable | Classification declaration |
| --- | --- |
| Season of conception | □Cold seasons (Nov. to Apr.)  □Warm seasons (May to Oct.) |
| Gravidity (times) | □ 1  □>1 |
| Parity | □Primiparous  □Multiparious |
| Lifestyle habits | |
| Maternal smoking | □Never  □Past  □Current |
| Paternal smoking | □Never  □Past  □Current |
| Passive smoking during pregnancy | □Yes  □No |
| Alcohol consumption during pregnancy | □Never  □Past  □Current |
| Folic acid and multivitamins before pregnancy | □None  □Folic acid or multivitamins  □Both |
| Folic acid and multivitamins during early pregnancy | □None  □Folic acid or multivitamins  □Both |
| Maternal medical conditions | |
| Maternal medical status before pregnancy  (Diabetes or hypertension) | □Yes  □No |

**Table S2 Information on Birth Outcomes**

| Type of variable | Classification declaration |
| --- | --- |
| Delivery information | |
| Date of birth (DOB) | Date: |
| Infant sex | □Boy  □Girl |
| Maternal medical condition during pregnancy  (Medical conditions during pregnancy refer to gestational complications, including gestational hypertension or gestational diabetes.) | □Yes  □No |

**
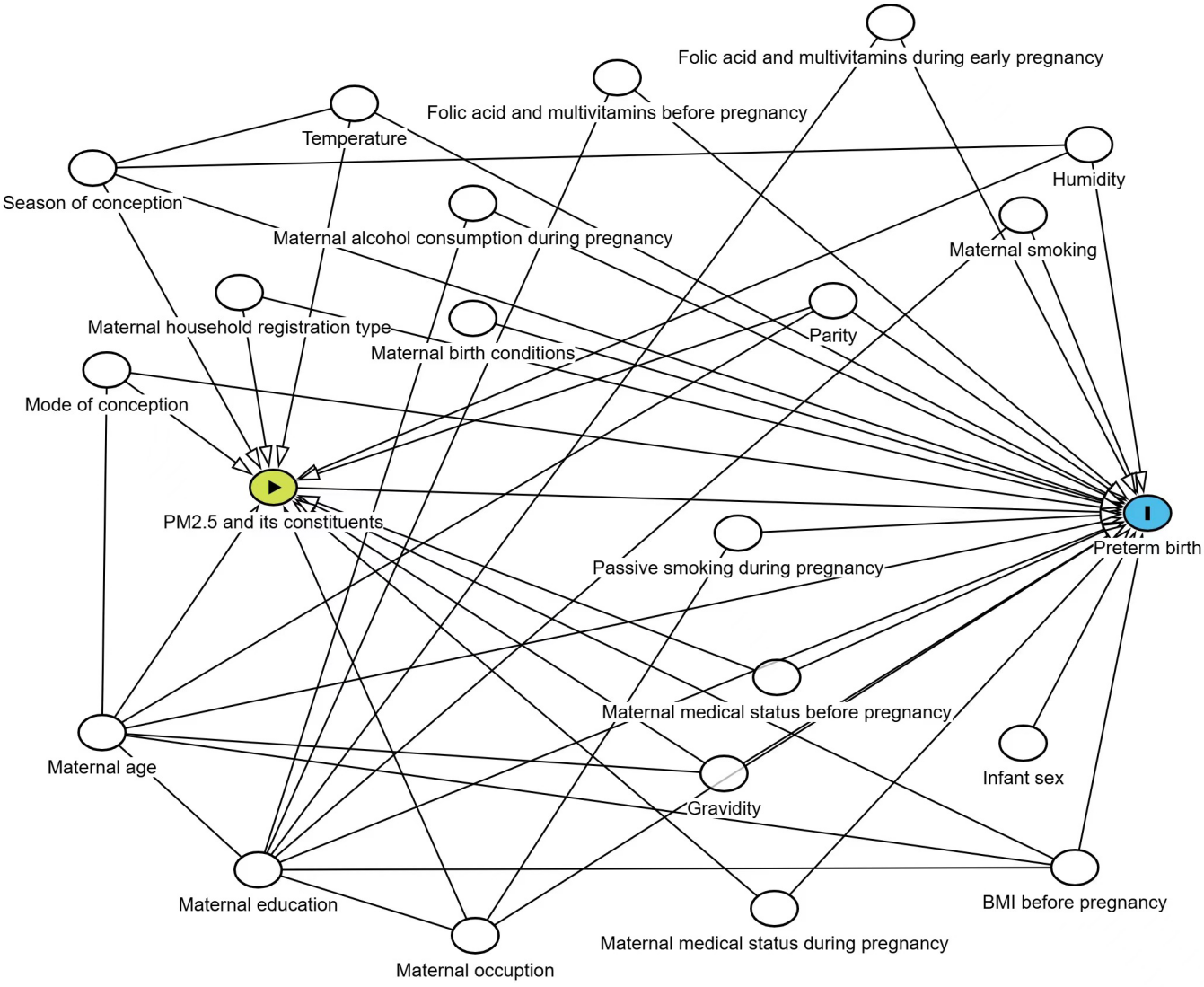
**

**Figure S1 shows the potential confounders using a Directed Acyclic Graph.**

**Table S3 Comparison of baseline demographic information between the study population and the source population**

| **Variables** | **Participants (%)** | **Source population (%)** | ***χ***2 | ***P*** |
| --- | --- | --- | --- | --- |
| N | 17240 | 27908 |  |  |
| Mother |  |  |  |  |
| **Household Registration Type** |  |  | 30.742 | <0.001*** |
| Local | 10462(60.68) | 16191(58.02) |  |  |
| Non-local | 6772(39.28) | 11696(41.91) |  |  |
| Missing | 6(0.03) | 21(0.08) |  |  |
| **Maternal age** |  |  | 46.557 | <0.001*** |
| <30 | 4652(26.98) | 8289(29.70) |  |  |
| 30-35 | 7793(45.20) | 12452(44.62) |  |  |
| >35 | 4795(27.81) | 7167(25.68) |  |  |
| Missing | 0(0.00) | 0(0.00) |  |  |
| **Maternal Education** |  |  | 6.567 | 0.087 |
| Middle school or below | 1061(6.15) | 1826(6.54) |  |  |
| Senior high school | 1608(9.33) | 2743(9.83) |  |  |
| College | 12319(71.46) | 19771(70.84) |  |  |
| Master or above | 2252(13.06) | 3564(12.77) |  |  |
| Missing | 0(0.00) | 4(0.01) |  |  |
| **Occupation** |  |  | 10.878 | 0.092 |
| Professional Technicians | 4372(25.36) | 6969(24.97) |  |  |
| Financial Services | 2759(16.00) | 4388(15.72) |  |  |
| Government official | 636(3.69) | 1006(3.60) |  |  |
| Service | 2122(12.31) | 3503(12.55) |  |  |
| Marketing | 1342(7.78) | 2004(7.18) |  |  |
| Media Advertising | 239(1.39) | 410(1.47) |  |  |
| Others | 5770(33.47) | 9625(34.49) |  |  |
| Missing | 0(0.00) | 3(0.01) |  |  |
| **BMI before pregnancy** |  |  | 3.623 | 0.163 |
| <18.5 | 2672(15.50) | 4513(16.17) |  |  |
| [18.5-24) | 12129(70.35) | 19483(69.81) |  |  |
| ≥24 | 2439(14.15) | 3909(14.01) |  |  |
| Missing | 0(0.00) | 3(0.01) |  |  |
| **Maternal Birth Conditions** |  |  | 0.151 | 0.927 |
| Term birth | 16498(95.70) | 26710(95.71) |  |  |
| Preterm birth | 438(2.54) | 695(2.49) |  |  |
| Post-term birth | 287(1.66) | 457(1.64) |  |  |
| Missing | 17(0.10) | 46(0.16) |  |  |

[continued]

Table S3 [continued]

| **Variables** | **Participants(%)** | **Source population (%)** | ***χ***2 | ***P*** |
| --- | --- | --- | --- | --- |
| **Mode of conception** |  |  | 37.603 | <0.001*** |
| Natural conception | 15678(90.94) | 24875(89.13) |  |  |
| Assisted Reproductive Techniques | 1561(9.05) | 3029(10.85) |  |  |
| Missing | 1(0.01) | 4(0.01) |  |  |
| **Season of conception** |  |  | 20.077 | <0.001*** |
| Cold seasons (Nov. to Apr.) | 7710(44.72) | 13086(46.89) |  |  |
| Warm seasons (May to Oct.) | 9530(55.28) | 14822(53.11) |  |  |
| Missing | 0(0.00) | 0(0.00) |  |  |
| **Gravidity** |  |  | 15.619 | <0.001*** |
| 1 | 6315(36.63) | 10742(38.49) |  |  |
| >1 | 10925(63.37) | 17166(61.51) |  |  |
| Missing | 0(0.00) | 0(0.00) |  |  |
| **Parity** |  |  | 34.767 | <0.001*** |
| Primiparous | 9511(55.17) | 16187(58.00) |  |  |
| Multiparious | 7729(44.83) | 11721(42.00) |  |  |
| Missing | 0(0.00) | 0(0.00) |  |  |
| **Maternal smoking** |  |  | 5.728 | 0.057 |
| Never | 16879(97.91) | 27226(97.56) |  |  |
| Past | 300(1.74) | 573(2.05) |  |  |
| Current | 61(0.35) | 106(0.38) |  |  |
| Missing | 0(0.00) | 3(0.01) |  |  |
| **Passive smoking during pregnancy** |  |  | 2.427 | 0.119 |
| No | 13711(79.53) | 22021(78.91) |  |  |
| Yes | 3528(20.46) | 5883(21.08) |  |  |
| Missing | 1(0.01) | 4(0.01) |  |  |
| **Alcohol consumption during pregnancy** |  |  | 1.523 | 0.467 |
| Never | 6770(39.27) | 10785(38.64) |  |  |
| Often | 652(3.78) | 1077(3.86) |  |  |
| Seldom | 9728(56.43) | 15861(56.83) |  |  |
| Missing | 90(0.52) | 185(0.66) |  |  |
| **Folic acid or multivitamins before pregnancy** |  |  | 1.382 | 0.501 |
| None | 6094(35.35) | 9713(34.80) |  |  |
| Folic acid or multivitamins | 8589(49.82) | 14029(50.27) |  |  |
| Both | 2557(14.83) | 4163(14.92) |  |  |
| Missing | 0(0.00) | 3(0.01) |  |  |
| **Folic acid or multivitamins during early pregnancy** |  |  | 0.567 | 0.753 |
| None | 1151(6.68) | 1852(6.64) |  |  |
| Folic acid or multivitamins | 11711(67.93) | 18878(67.64) |  |  |
| Both | 4378(25.39) | 7175(25.71) |  |  |
| Missing | 0(0.00) | 3(0.01) |  |  |

[continued]

Table S3 [continued]

| **Variables** | **Participants(%)** | **Source population (%)** | ***χ***2 | ***P*** |
| --- | --- | --- | --- | --- |
| **Maternal medical status before pregnancy** |  |  | 0.650 | 0.420 |
| No | 17041(98.85) | 27591(98.86) |  |  |
| Yes | 136(0.79) | 200(0.72) |  |  |
| Missing | 63(0.37) | 117(0.42) |  |  |

**Note:** BMI, Body mass index. N(%): numbers and proportion. **p*<0.05; ***p*<0.01; ****p*<0.001.

**Table S4 Summary statistics of exposure level to air pollutants and ambient temperature**

**at different stages of pregnancy**

| **Air pollutants** |  | **Percentile** | | | | | **Max** | **Mean** | **SD** |
| --- | --- | --- | --- | --- | --- | --- | --- | --- | --- |
|  | **Min** | **5_th_** | **25_th_** | **50_th_** | **75_th_** | **95_th_** |  |  |  |
| **Trimester 1** |  |  |  |  |  |  |  |  |  |
| PM2.5 (μg/m^3^) | 4.76 | 11.21 | 16.20 | 21.01 | 25.03 | 31.86 | 41.53 | 20.93 | 6.15 |
| SO42- (μg/m^3^) | 0.99 | 2.11 | 3.21 | 4.18 | 4.94 | 6.42 | 8.06 | 4.16 | 1.27 |
| NO3- (μg/m^3^) | 0.57 | 0.95 | 1.43 | 2.09 | 3.34 | 4.35 | 10.52 | 2.39 | 1.12 |
| NH4+ (μg/m^3^) | 0.52 | 0.82 | 1.16 | 1.60 | 2.30 | 2.86 | 6.79 | 1.73 | 0.67 |
| BC (μg/m^3^) | 0.32 | 0.63 | 0.92 | 1.19 | 1.43 | 1.89 | 2.26 | 1.20 | 0.37 |
| OM (μg/m^3^) | 1.40 | 3.05 | 4.52 | 5.97 | 7.15 | 9.34 | 11.44 | 5.96 | 1.86 |
| Temperature (℃) | 8.22 | 15.92 | 17.96 | 21.92 | 26.29 | 27.88 | 28.48 | 21.97 | 4.26 |
| Relative Humidity (%) | 57.86 | 68.76 | 72.14 | 76.87 | 83.37 | 86.42 | 88.31 | 77.39 | 5.98 |
| **Trimester 2** |  |  |  |  |  |  |  |  |  |
| PM2.5 (μg/m^3^) | 5.36 | 11.20 | 16.14 | 21.27 | 24.94 | 31.49 | 63.57 | 20.92 | 6.04 |
| SO42- (μg/m^3^) | 1.14 | 2.12 | 3.17 | 4.22 | 4.94 | 6.34 | 11.71 | 4.15 | 1.24 |
| NO3- (μg/m^3^) | 0.66 | 0.95 | 1.47 | 2.14 | 3.37 | 4.31 | 15.55 | 2.41 | 1.10 |
| NH4+ (μg/m^3^) | 0.60 | 0.82 | 1.17 | 1.63 | 2.32 | 2.85 | 9.47 | 1.74 | 0.67 |
| BC (μg/m^3^) | 0.36 | 0.63 | 0.91 | 1.20 | 1.42 | 1.87 | 2.97 | 1.20 | 0.36 |
| OM (μg/m^3^) | 1.57 | 3.05 | 4.53 | 6.04 | 7.10 | 9.19 | 16.60 | 5.95 | 1.82 |
| Temperature (℃) | 10.47 | 15.93 | 17.75 | 21.32 | 25.80 | 27.84 | 28.59 | 21.69 | 4.18 |
| Relative Humidity (%) | 61.26 | 68.17 | 72.07 | 76.99 | 83.34 | 86.00 | 88.01 | 77.30 | 5.98 |
| **Trimester 3** |  |  |  |  |  |  |  |  |  |
| PM2.5 (μg/m^3^) | 5.53 | 10.43 | 13.76 | 18.81 | 22.88 | 27.23 | 44.28 | 18.57 | 5.44 |
| SO42- (μg/m^3^) | 1.12 | 1.98 | 2.65 | 3.76 | 4.47 | 5.36 | 9.93 | 3.65 | 1.12 |
| NO3- (μg/m^3^) | 0.52 | 0.89 | 1.23 | 1.77 | 2.44 | 3.97 | 6.53 | 2.00 | 0.97 |
| NH4+ (μg/m^3^) | 0.45 | 0.75 | 0.99 | 1.38 | 1.85 | 2.68 | 4.72 | 1.49 | 0.61 |
| BC (μg/m^3^) | 0.35 | 0.59 | 0.77 | 1.06 | 1.28 | 1.57 | 2.87 | 1.05 | 0.32 |
| OM (μg/m^3^) | 1.57 | 2.90 | 3.83 | 5.29 | 6.48 | 7.82 | 13.35 | 5.24 | 1.59 |
| Temperature (℃) | 11.30 | 15.62 | 19.17 | 24.37 | 27.13 | 28.08 | 29.23 | 23.13 | 4.32 |
| Relative Humidity (%) | 55.33 | 67.78 | 74.61 | 80.61 | 85.14 | 86.53 | 90.92 | 79.40 | 6.19 |
| **Entire pregnancy** |  |  |  |  |  |  |  |  |  |
| PM2.5 (μg/m^3^) | 10.64 | 16.24 | 18.30 | 20.36 | 22.11 | 24.06 | 46.30 | 20.24 | 2.50 |
| SO42- (μg/m^3^) | 2.19 | 3.22 | 3.61 | 4.00 | 4.39 | 4.80 | 8.66 | 4.00 | 0.50 |
| NO3- (μg/m^3^) | 1.06 | 1.56 | 2.01 | 2.39 | 2.57 | 2.78 | 9.78 | 2.28 | 0.40 |
| NH4+ (μg/m^3^) | 0.91 | 1.22 | 1.49 | 1.72 | 1.84 | 1.99 | 6.09 | 1.67 | 0.25 |
| BC (μg/m^3^) | 0.63 | 0.92 | 1.04 | 1.15 | 1.27 | 1.40 | 2.32 | 1.15 | 0.15 |
| OM (μg/m^3^) | 2.98 | 4.57 | 5.18 | 5.78 | 6.30 | 6.89 | 12.68 | 5.75 | 0.73 |
| Temperature (℃) | 15.35 | 20.49 | 21.09 | 21.86 | 23.39 | 24.59 | 26.79 | 22.21 | 1.37 |
| Relative Humidity (%) | 67.93 | 74.37 | 76.44 | 77.83 | 79.90 | 81.24 | 84.81 | 77.94 | 2.23 |

**Note:** PM_2.5_, particulate matter with an aerodynamic diameter ≤ 2.5 µm; BC, Black carbon; NH_4_^+^, ammonium; NO_3_^-^, Nitrate; OM, Organic matter; SO_4_^2-^: Sulfate; SD: standard deviation.

**Table S5 Odds ratios (95% CI) of PTB per IQR increase in PM_2.5_ and its constituents concentrations**

**in each trimester and the whole pregnancy in different models (*df*=3)**

| **Air pollutants** | ***Model I*** | | |  | ***Model II*** | | |  | ***Model III*** | | |
| --- | --- | --- | --- | --- | --- | --- | --- | --- | --- | --- | --- |
|  | **OR (95%*CI*)** | ***P*** Value | **AIC** |  | **OR (95%*CI*)** | ***P*** Value | **AIC** |  | **OR (95%*CI*)** | ***P*** Value | **AIC** |
| **PM_2.5_** |  |  |  |  |  |  |  |  |  |  |  |
| Trimester 1 | 0.85(0.70,1.05) | 0.133 | 7815.467 |  | 0.90(0.73,1.10) | 0.301 | 7729.626 |  | 0.92(0.74,1.13) | 0.408 | 7664.516 |
| Trimester 2 | 0.91(0.74,1.11) | 0.353 | 7812.594 |  | 0.94(0.76,1.15) | 0.527 | 7727.001 |  | 0.96(0.78,1.18) | 0.688 | 7664.062 |
| Trimester 3 | 2.03(1.60,2.57) | <0.001*** | 7517.276 |  | 1.96(1.55,2.49) | <0.001*** | 7432.670 |  | 2.54(1.99,3.24) | <0.001*** | 7321.560 |
| Entire pregnancy | 0.90(0.76,1.08) | 0.258 | 6850.990 |  | 0.93(0.78,1.11) | 0.431 | 6811.941 |  | 0.99(0.83,1.19) | 0.936 | 6747.784 |
| **BC** |  |  |  |  |  |  |  |  |  |  |  |
| Trimester 1 | 0.85(0.71,1.01) | 0.066 | 7814.338 |  | 0.88(0.74,1.05) | 0.169 | 7728.802 |  | 0.89(0.74,1.07) | 0.217 | 7663.699 |
| Trimester 2 | 0.90(0.75,1.08) | 0.250 | 7812.118 |  | 0.93(0.77,1.11) | 0.401 | 7726.682 |  | 0.95(0.79,1.14) | 0.561 | 7663.876 |
| Trimester 3 | 1.90(1.53,2.36) | <0.001*** | 7518.981 |  | 1.84(1.48,2.28) | <0.001*** | 7434.556 |  | 2.25(1.81,2.81) | <0.001*** | 7325.755 |
| Entire pregnancy | 0.83(0.70,0.98) | 0.029* | 6847.495 |  | 0.86(0.72,1.01) | 0.070 | 6809.273 |  | 0.91(0.76,1.08) | 0.288 | 6746.654 |
| **NH_4_^+^** |  |  |  |  |  |  |  |  |  |  |  |
| Trimester 1 | 0.95(0.66,1.35) | 0.766 | 7817.499 |  | 1.00(0.70,1.42) | 0.982 | 7730.472 |  | 1.07(0.74,1.54) | 0.724 | 7664.789 |
| Trimester 2 | 0.93(0.65,1.33) | 0.677 | 7813.142 |  | 0.93(0.65,1.34) | 0.709 | 7727.110 |  | 0.97(0.68,1.38) | 0.856 | 7664.092 |
| Trimester 3 | 1.38(1.05,1.83) | 0.022* | 7546.106 |  | 1.33(1.01,1.75) | 0.042* | 7460.255 |  | 1.39(1.05,1.82) | 0.020* | 7369.491 |
| Entire pregnancy | 1.14(0.92,1.42) | 0.240 | 6850.884 |  | 1.16(0.93,1.45) | 0.191 | 6810.860 |  | 1.26(1.00,1.59) | 0.046* | 6743.843 |
| **NO_3_^-^** |  |  |  |  |  |  |  |  |  |  |  |
| Trimester 1 | 0.79(0.57,1.09) | 0.154 | 7816.416 |  | 0.83(0.60,1.15) | 0.269 | 7729.912 |  | 0.91(0.64,1.29) | 0.594 | 7664.935 |
| Trimester 2 | 0.93(0.66,1.31) | 0.667 | 7813.079 |  | 0.95(0.68,1.33) | 0.758 | 7727.126 |  | 0.98(0.70,1.38) | 0.926 | 7664.097 |
| Trimester 3 | 1.16(0.92,1.47) | 0.219 | 7549.804 |  | 1.14(0.90,1.45) | 0.264 | 7463.109 |  | 1.11(0.88,1.41) | 0.386 | 7374.079 |
| Entire pregnancy | 1.59(1.26,2.02) | 0.001*** | 6837.718 |  | 1.64(1.29,2.08) | <0.001*** | 6796.406 |  | 1.82(1.42,2.33) | <0.001*** | 6725.266 |

[continued]

Table S5 [continued]

| **Air pollutants** | ***Model I*** | | |  | ***Model II*** | | |  | ***Model III*** | | |
| --- | --- | --- | --- | --- | --- | --- | --- | --- | --- | --- | --- |
|  | **OR (95%*CI*)** | ***P*** Value | **AIC** |  | **OR (95%*CI*)** | ***P*** Value | **AIC** |  | **OR (95%*CI*)** | ***P*** Value | **AIC** |
| **OM** |  |  |  |  |  |  |  |  |  |  |  |
| Trimester 1 | 0.84(0.69,1.02) | 0.081 | 7814.677 |  | 0.88(0.72,1.07) | 0.199 | 7729.045 |  | 0.89(0.73,1.09) | 0.258 | 7663.936 |
| Trimester 2 | 0.92(0.76,1.11) | 0.386 | 7812.699 |  | 0.95(0.78,1.15) | 0.569 | 7727.071 |  | 0.97(0.80,1.18) | 0.745 | 7664.117 |
| Trimester 3 | 1.98(1.57,2.51) | <0.001*** | 7519.180 |  | 1.92(1.52,2.42) | <0.001*** | 7434.697 |  | 2.42(1.91,3.08) | <0.001*** | 7325.015 |
| Entire pregnancy | 0.85(0.72,1.01) | 0.070 | 6848.968 |  | 0.88(0.74,1.05) | 0.149 | 6810.477 |  | 0.94(0.78,1.12) | 0.485 | 6747.299 |
| **SO_4_^2-^** |  |  |  |  |  |  |  |  |  |  |  |
| Trimester 1 | 0.87(0.73,1.03) | 0.115 | 7815.164 |  | 0.90(0.76,1.07) | 0.243 | 7729.283 |  | 0.91(0.76,1.09) | 0.293 | 7664.071 |
| Trimester 2 | 0.94(0.78,1.12) | 0.468 | 7812.948 |  | 0.96(0.80,1.15) | 0.647 | 7727.196 |  | 0.98(0.82,1.17) | 0.832 | 7664.186 |
| Trimester 3 | 1.06(1.66,2.56) | <0.001*** | 7508.835 |  | 1.99(1.60,2.47) | <0.001*** | 7425.603 |  | 2.53(2.03,3.15) | <0.001*** | 7311.054 |
| Entire pregnancy | 0.71(0.59,0.85) | <0.001*** | 6838.180 |  | 0.73(0.61,0.88) | <0.001 *** | 6801.147 |  | 0.79(0.65,0.95) | 0.012* | 6741.385 |

**Note:** PM_2.5_, particulate matter with an aerodynamic diameter ≤ 2.5 µm; BC, Black carbon; NH_4_^+^, ammonium; NO_3_^-^, Nitrate; OM, Organic matter; SO_4_^2-^: Sulfate. Model I, a crude model, just includes the concentration of PM2.5. Model II additionally adjusts for demographic information, including household registration type, maternal education, occupation, maternal age, BMI before pregnancy, maternal birth conditions, and maternal medical status before pregnancy. Model III further adjusts for mode of conception, season of conception, gravidity, parity, alcohol consumption during pregnancy, maternal smoking, passive smoking during pregnancy, folic acid and multivitamins before pregnancy, folic acid and multivitamins during early pregnancy, maternal medical status during pregnancy, and infant sex. The above models were all adjusted for temperature and humidity. **p*<0.05, ***p*<0.01, ****p*<0.001.

**Table S6 Odds ratios (95% CI) of PTB per IQR increase in PM_2.5_ and its constituents concentrations**

**in each trimester and the whole pregnancy in different models (*df*=4)**

| **Air pollutants** | ***Model I*** | | |  | ***Model II*** | | |  | ***Model III*** | | |
| --- | --- | --- | --- | --- | --- | --- | --- | --- | --- | --- | --- |
|  | **OR (95%*CI*)** | ***P* Value** | **AIC** |  | **OR (95%*CI*)** | ***P* Value** | **AIC** |  | **OR (95%*CI*)** | ***P* Value** | **AIC** |
| **PM_2.5_** |  |  |  |  |  |  |  |  |  |  |  |
| Trimester 1 | 0.85(0.70-1.05) | 0.133 | 7815.500 |  | 0.90(0.73,1.10) | 0.298 | 7729.685 |  | 0.92(0.75，1.14) | 0.463 | 7664.025 |
| Trimester 2 | 0.91(0.74,1.12) | 0.368 | 7812.190 |  | 0.94(0.76-1.15) | 0.539 | 7726.885 |  | 0.96(0.78，1.18) | 0.701 | 7663.966 |
| Trimester 3 | 1.99(1.57,2.53) | <0.001*** | 7518.532 |  | 1.94(1.53-2.46) | <0.001*** | 7433.960 |  | 2.49(1.95，3.18) | <0.001*** | 7317.157 |
| Entire pregnancy | 0.98(0.82,1.17) | 0.827 | 6782.890 |  | 1.01(0.84,1.21) | 0.917 | 6744.498 |  | 1.05(0.88,1.27) | 0.585 | 6681.526 |
| **BC** |  |  |  |  |  |  |  |  |  |  |  |
| Trimester 1 | 0.85(0.71,1.01) | 0.066 | 7814.392 |  | 0.88(0.74,1.05) | 0.168 | 7728.877 |  | 0.90(0.75,1.08) | 0.245 | 7663.257 |
| Trimester 2 | 0.90(0.75,1.08) | 0.252 | 7811.652 |  | 0.93(0.77,1.11) | 0.404 | 7726.546 |  | 0.95(0.79,1.14) | 0.566 | 7663.772 |
| Trimester 3 | 1.86(1.49,2.31) | <0.001*** | 7520.401 |  | 1.81(1.45-2.25) | <0.001*** | 7436.048 |  | 2.20(1.76,2.75) | <0.001*** | 7321.722 |
| Entire pregnancy | 0.90(0.76,1.07) | 0.246 | 6781.649 |  | 0.93(0.78，1.11） | 0.414 | 6743.888 |  | 0.98(0.82,1.17) | 0.821 | 6681.802 |
| **NH_4_^+^** |  |  |  |  |  |  |  |  |  |  |  |
| Trimester 1 | 0.95(0.67,1.36) | 0.796 | 7817.492 |  | 0.99(0.70,1.41) | 0.964 | 7730.547 |  | 1.13(0.77,0.164) | 0.536 | 7659.592 |
| Trimester 2 | 0.93(0.65,1.34) | 0.704 | 7812.719 |  | 0.94(0.66,1.35) | 0.745 | 7727.007 |  | 0.97(0.68,1.39) | 0.881 | 7659.710 |
| Trimester 3 | 1.38(1.05,1.82) | 0.022 | 7546.359 |  | 1.33(1.01,1.76) | 0.040 | 7460.898 |  | 1.44(1.07,1.93) | 0.015 | 7357.662 |
| Entire pregnancy | 1.25(1.00,1.56) | 0.051 | 6779.076 |  | 1.28(1.02,1.60) | 0.035 | 6740.014 |  | 1.39(1.10,1.75) | 0.005** | 6673.988 |
| **NO_3_^-^** |  |  |  |  |  |  |  |  |  |  |  |
| Trimester 1 | 0.79(0.57,1.09) | 0.147 | 7816.424 |  | 0.83(0.60,1.14) | 0.252 | 7729.946 |  | 0.67(0.97,1.40) | 0.863 | 7664.418 |
| Trimester 2 | 0.93(0.66,1.32) | 0.693 | 7812.655 |  | 0.96(0.68,1.34) | 0.799 | 7727.011 |  | 0.99(0.71,1.39) | 0.963 | 7663.991 |
| Trimester 3 | 1.17(0.92,1.48) | 0.193 | 7549.822 |  | 1.15(0.91,1.46) | 0.236 | 7463.612 |  | 1.14(0.86,1.50) | 0.358 | 7366.028 |
| Entire pregnancy | 1.80(1.42,2.29) | <0.001*** | 6759.951 |  | 1.86(1.46,2.37) | <0.001*** | 6719.397 |  | 2.07(1.62,2.65) | <0.001*** | 6648.854 |

[continued]

Table S6 [continued]

| **Air pollutants** | ***Model I*** | | |  | ***Model II*** | | |  | ***Model III*** | | |
| --- | --- | --- | --- | --- | --- | --- | --- | --- | --- | --- | --- |
|  | **OR (95%*CI*)** | ***P* Value** | **AIC** |  | **OR (95%*CI*)** | ***P* Value** | **AIC** |  | **OR (95%*CI*)** | ***P* Value** | **AIC** |
| **OM** |  |  |  |  |  |  |  |  |  |  |  |
| Trimester 1 | 0.84(0.69,1.02) | 0.081 | 7814.711 |  | 0.88(0.72,1.07) | 0.198 | 7729.113 |  | 0.90(0.74,1.11) | 0.290 | 7663.466 |
| Trimester 2 | 0.92(0.76,1.11) | 0.393 | 7812.244 |  | 0.95(0.78,1.15) | 0.575 | 7726.934 |  | 0.97(0.80,1.18) | 0.752 | 7664.005 |
| Trimester 3 | 1.94(1.53,2.45) | <0.001*** | 7520.550 |  | 1.88(1.49,2.38) | <0.001*** | 7436.093 |  | 2.37(1.86,3.02) | <0.001*** | 7320.468 |
| Entire pregnancy | 0.93(0.77,1.11) | 0.398 | 6782.248 |  | 0.96(0.80,1.14) | 0.618 | 6744.283 |  | 0.97(0.80,1.18) | 0.752 | 6681.841 |
| **SO_4_^2-^** |  |  |  |  |  |  |  |  |  |  |  |
| Trimester 1 | 0.87(0.73,1.03) | 0.116 | 7815.232 |  | 0.90(0.76,1.07) | 0.243 | 7729.366 |  | 0.91(0.77,1.09) | 0.324 | 7663.598 |
| Trimester 2 | 0.94(0.78,1.12) | 0.478 | 7812.483 |  | 0.96(0.80,1.15) | 0.653 | 7727.058 |  | 0.98(0.82,1.18) | 0.840 | 7664.070 |
| Trimester 3 | 2.02(1.62,2.51) | <0.001*** | 7510.330 |  | 1.95(1.57,2.42) | <0.001*** | 7427.113 |  | 2.47(1.98,3.09) | <0.001*** | 7306.954 |
| Entire pregnancy | 0.77(0.64,0.92) | 0.005** | 6775.040 |  | 0.79(0.66,0.95) | 0.01328* | 6738.474 |  | 0.84(0.69,1.02) | 0.073 | 6678.683 |

**Note:** PM_2.5_, particulate matter with an aerodynamic diameter ≤ 2.5 µm; BC, Black carbon; NH_4_^+^, ammonium; NO_3_^-^, Nitrate; OM, Organic matter; SO_4_^2-^: Sulfate. Model I, a crude model, just includes the concentration of PM2.5. Model II additionally adjusts for demographic information, including household registration type, maternal education, occupation, maternal age, BMI before pregnancy, maternal birth conditions, and maternal medical status before pregnancy. Model III further adjusts for mode of conception, season of conception, gravidity, parity, alcohol consumption during pregnancy, maternal smoking, passive smoking during pregnancy, folic acid and multivitamins before pregnancy, folic acid and multivitamins during early pregnancy, maternal medical status during pregnancy, and infant sex. The above models were all adjusted for temperature and humidity*.* **p*<0.05, ***p*<0.01, ****p*<0.001.

**Table S7 The results of the AIC for cubic splines with different degrees of freedom**

**in Model III at different stages of pregnancy**

| **Periods of pregnancy** | **Akaike Information Criterion** | | |
| --- | --- | --- | --- |
|  | ***df***=3 | ***df***=4 | ***df***=5 |
| **Trimester 1** |  |  |  |
| PM_2.5_ | 7664.516 | 7664.025 | 7663.254 |
| BC | 7663.699 | 7663.257 | 7662.408 |
| OM | 7663.936 | 7663.466 | 7662.682 |
| NO_3_^-^ | 7664.935 | 7664.418 | 7663.692 |
| SO_4_^2-^ | 7664.071 | 7663.598 | 7662.762 |
| NH_4_^+^ | 7664.789 | 7663.865 | 7663.045 |
| **Trimester 2** |  |  |  |
| PM_2.5_ | 7664.062 | 7663.966 | 7664.086 |
| BC | 7663.876 | 7663.772 | 7663.895 |
| OM | 7664.117 | 7664.005 | 7664.127 |
| NO_3_^-^ | 7664.097 | 7663.991 | 7664.114 |
| SO_4_^2-^ | 7664.186 | 7664.070 | 7664.190 |
| NH_4_^+^ | 7664.092 | 7663.992 | 7664.114 |
| **Trimester 3** |  |  |  |
| PM_2.5_ | 7321.560 | 7317.157 | 7208.622 |
| BC | 7325.755 | 7321.722 | 7218.063 |
| OM | 7325.015 | 7320.468 | 7215.114 |
| NO_3_^-^ | 7374.079 | 7366.028 | 7278.624 |
| SO_4_^2-^ | 7311.054 | 7306.954 | 7202.829 |
| NH_4_^+^ | 7369.491 | 7361.607 | 7271.407 |
| **The entire pregnancy** |  |  |  |
| PM_2.5_ | 6747.784 | 6681.526 | 6412.878 |
| BC | 6746.654 | 6681.802 | 6414.099 |
| OM | 6747.299 | 6681.841 | 6414.200 |
| NO_3_^-^ | 6725.266 | 6648.854 | 6409.861 |
| SO_4_^2-^ | 6741.385 | 6678.683 | 6411.293 |
| NH_4_^+^ | 6743.843 | 6673.988 | 6414.212 |

**Note:** AIC, Akaike Information Criterion; PM_2.5_, particulate matter with an aerodynamic diameter ≤ 2.5 µm; BC, Black carbon; NH_4_^+^, ammonium; NO_3_^-^, Nitrate; OM, Organic matter; SO_4_^2-^: Sulfate.


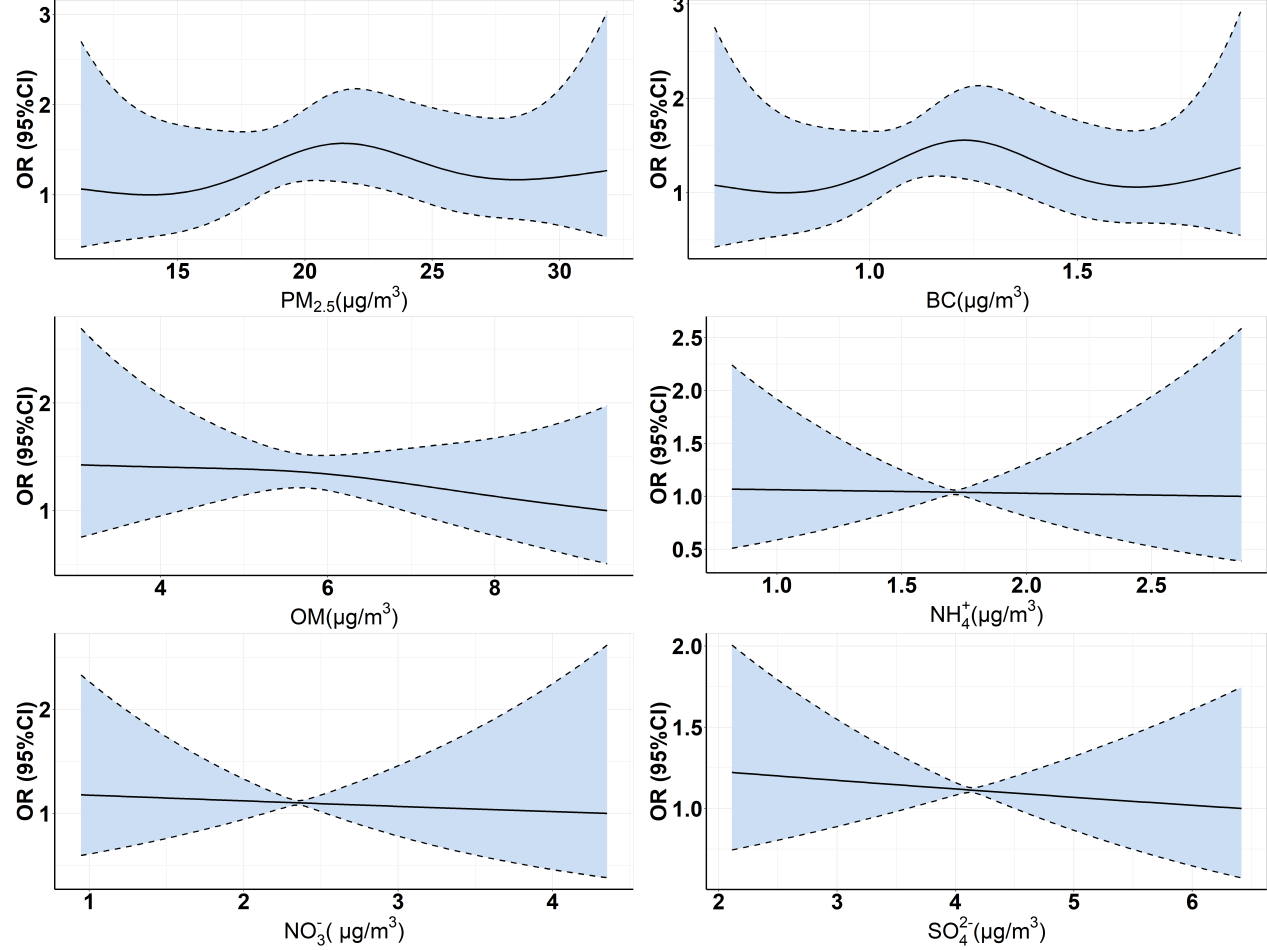


**Figure S2** shows the non-linear association between PM_2.5_ exposure and its constituents

with PTB in 1^st^ trimester.


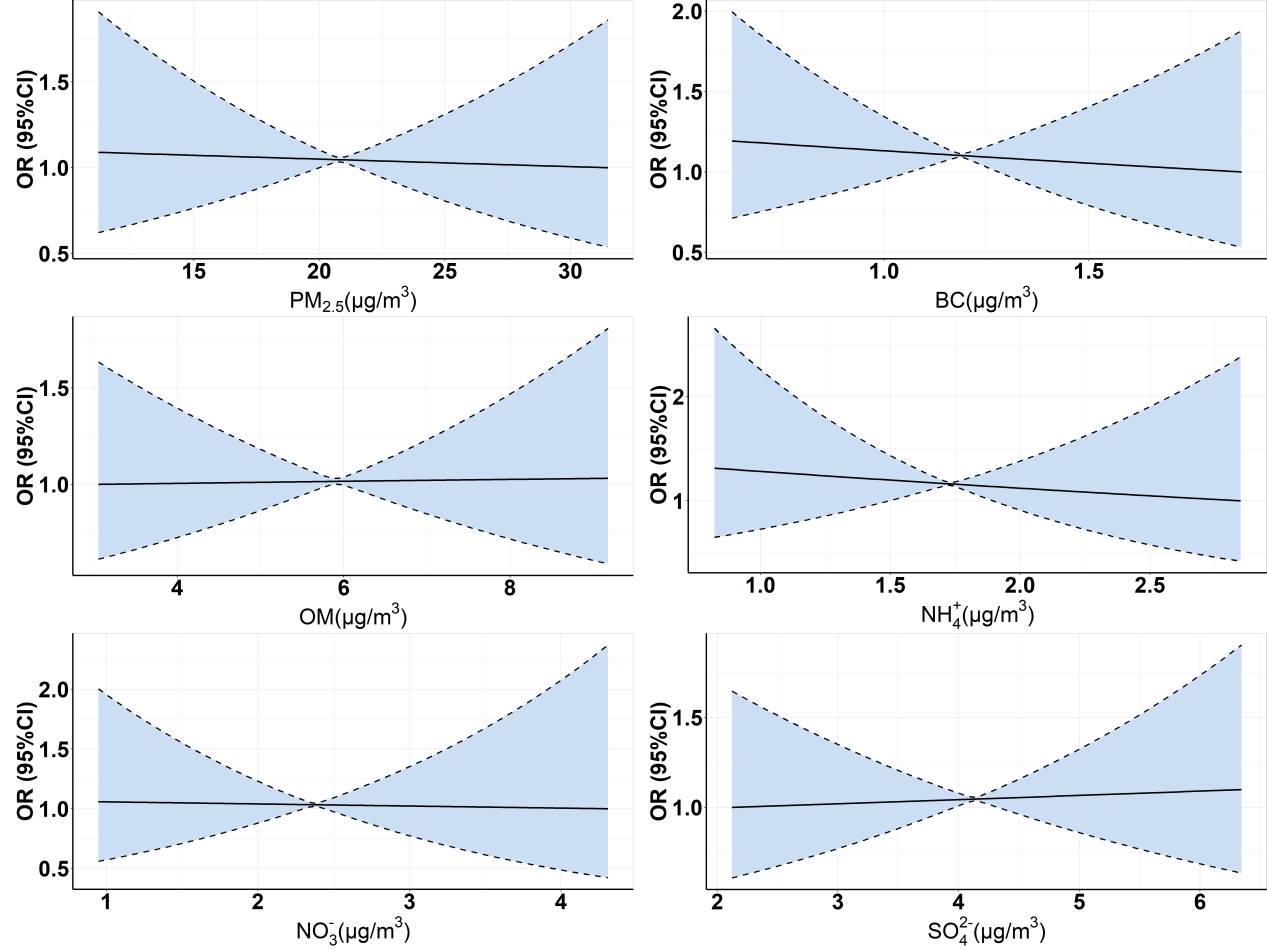


**Figure S3** shows the non-linear association between PM_2.5_ exposure and its constituents

with PTB in 2^nd^ trimester.


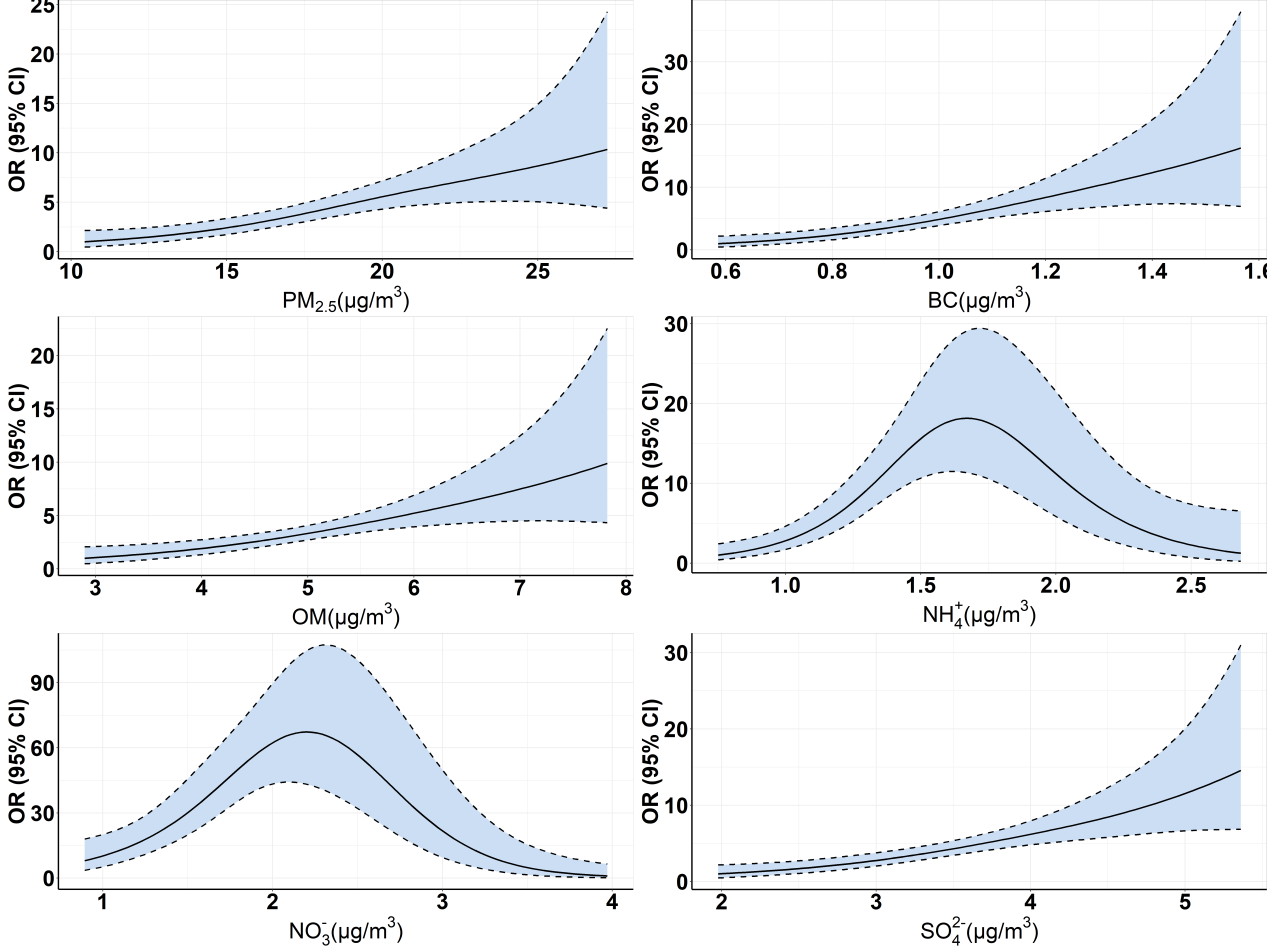


**Figure S4** shows the non-linear association between PM_2.5_ exposure and its constituents

with PTB in 3^rd^ trimester.


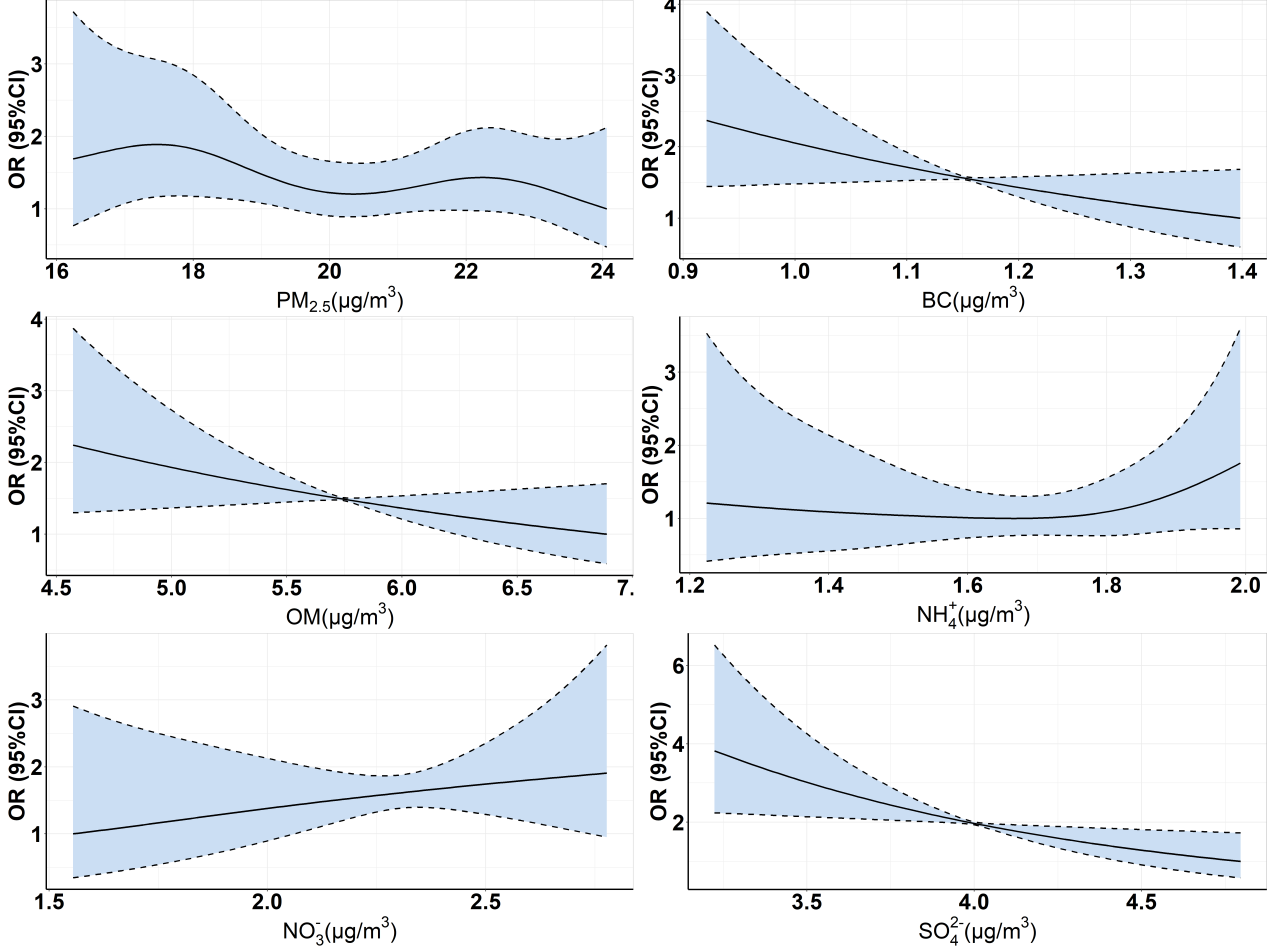


**Figure S5** shows the non-linear association between PM_2.5_ exposure and its constituents

with PTB in the entire pregnancy.

**Table S8 Odds ratios (95% *CI*) of PTB per IQR increase in PM_2.5_ and constituents concentrations in each trimester and the whole pregnancy in *model III* (*df=5*)**

| **Periods of pregnancy** | **OR (95% CI)** | | | | | |
| --- | --- | --- | --- | --- | --- | --- |
|  | **PM_2.5_** | **BC** | **NH_4_^+^** | **NO_3_^-^** | **OM** | **SO_4_^2-^** |
| **Trimester 1** | 0.94(0.76,1.16) | 0.91(0.75,1.09) | 1.19(0.81,1.74) | 1.03(0.71,1.50) | 0.91(0.74,1.11) | 0.92(0.77,1.10) |
| **Trimester 2** | 0.97(0.79,1.19) | 0.95(0.79,1.15) | 0.99(0.69,1.42) | 1.01(0.72,1.41) | 0.98(0.80,1.19) | 0.99(0.82,1.18) |
| **Trimester 3** | 3.29(2.53,4.27)*** | 2.76(2.18,3.49)*** | 1.78(1.31,2.42)*** | 1.44(1.07,1.92)* | 3.03(2.35,3.91)  *** | 3.09(2.44,3.91)*** |
| **Entire pregnancy** | 1.12(0.93,1.35) | 0.97(0.81,1.17) | 1.01(0.79,1.30) | 1.37(1.05,1.79)* | 1.01(0.84,1.23) | 0.85(0.70,1.03) |

**Note:** Sensitivity analysis, building on *Model III (df=5)*, further adjusts for the husband's household registration type, education level, age, and smoking status. PM_2.5_, particulate matter with an aerodynamic diameter ≤ 2.5 µm; BC, Black carbon; NH_4_^+^, ammonium; NO_3_^-^, Nitrate; OM, Organic matter; SO_4_^2-^: Sulfate. **p*<0.05; ***p*<0.01; ****p*<0.001.

**Table S9 Odds ratios (95% CI) of PTB per IQR increase in PM_2.5_ and its constituents concentrations in each trimester and the whole pregnancy in *model III* (**df**=5)**

| **Periods of pregnancy** | **OR (95% CI)** | | | | | |
| --- | --- | --- | --- | --- | --- | --- |
|  | **PM_2.5_** | **BC** | **NH_4_^+^** | **NO_3_^-^** | **OM** | **SO_4_^2-^** |
| **Trimester 1** | 0.92(0.74,1.14) | 0.90(0.74,1.08) | 1.11(0.75,1.63) | 0.95(0.65,1.40) | 0.90(0.73,1.10) | 0.92(0.77,1.10) |
| **Trimester 2** | 0.93(0.76,1.15) | 0.92(0.76,1.11) | 0.97(0.67,1.39) | 0.98(0.69,1.38) | 0.94(0.77,1.14) | 0.95(0.79,1.15) |
| **Trimester 3** | 3.20(2.46,4.14)*** | 2.69(2.12,3.40)*** | 1.74(1.28,2.36)*** | 1.40(1.05,1.87)* | 2.95(2.29,3.79)  *** | 3.00(2.38,3.80)  *** |
| **Entire pregnancy** | 1.06(0.88,1.28) | 0.92(0.77,1.11) | 0.92(0.72,1.19) | 1.22(0.93,1.60) | 0.96(0.80,1.16) | 0.80(0.66,0.98)* |

**Note:** Sensitivity analysis excluded pregnant women with gestational age less than 28 weeks and removed the "infant sex" covariate from the model. PM_2.5_, particulate matter with an aerodynamic diameter ≤ 2.5 µm; BC, Black carbon; NH_4_^+^, ammonium; NO_3_^-^, Nitrate; OM, Organic matter; SO_4_^2-^: Sulfate, PTB: Preterm birth. **p*<0.05; ***p*<0.01; ****p*<0.001.
